# Quantitative Structure-Activity Relationship Model to Predict Antioxidant Effects of the Peptide Fraction Extracted from a Co-Culture System of *Chlorella pyrenoidosa* and *Yarrowia lipolytica*

**DOI:** 10.3390/md17110633

**Published:** 2019-11-08

**Authors:** Huifan Liu, Sufen Li, Yuming Zhong, Jianliang Liu, Hui Liu, Jian Cheng, Lukai Ma, Yuqing Huang, Xuanyi Cai, Haijun Liu, Jiantong Zheng, Zhongai Su, Qin Wang

**Affiliations:** Zhongkai University of Agriculture and Engineering, Guangzhou, Guangdong 510225, China; lm_zkng@163.com (H.L.); m199lk@163.com (L.M.); Whitesue@163.com (Z.S.)

**Keywords:** *Chlorella pyrenoidosa*, *Yarrowia lipolytica*, enzymatic hydrolysis peptides, HepG2, quantitative structure–activity relationship model

## Abstract

In this study, the antioxidant components in co-culture of *Chlorella pyrenoidosa* and *Yarrowia lipolytica* (3:1 ratio) were confirmed as trypsin-hydrolyzed peptides (EHPs). The EHPs were composed of 836 different peptides with molecular weights ranging from 639 to 3531 Da and were mainly composed of hydrophobic amino acids (48.1%). These peptides showed remarkable protective effects against oxidative stress in HepG2, which may be attributed to their structures. Furthermore, the mRNA and protein levels of nuclear factor erythroid 2-related factor 2 (Nrf2) were significantly lower in the peptide-treated group than in the control group, suggesting that the antioxidant enzyme-coding genes were not activated. The EC_50_ value of three peptides in the EHPs were in the order of AGYSPIGFVR (0.04 ± 0.002 mg/mL) > VLDELTLAR (0.09 ± 0.001 mg/mL) > LFDPVYLFDQG (0.41 ± 0.03 mg/mL); these results agreed with the prediction of the model (*R*^2^ > 0.9, Q^2^ > 0.5). Thus, EHPs show potential as potent new antioxidant agents.

## 1. Introduction

*Chlorella pyrenoidesa* is a ubiquitous mononuclear marine alga that depends on photosynthesis and autotrophy for its growth and propagation [1]. In particular, the protein content of *C. pyrenoidesa* can reach up to 40% and the proteins consist of 17 types of amino acids [2]. The proteins are well-known to have antioxidative [3] and anti-tumor [4] properties. *Chlorella pyrenoidesa* has been shown to benefit from symbiotic co-culture with various bacteria, which improves its photosynthetic activity and growth [5]. *Chlorella pyrenoidesa* provides oxygen for the metabolism of the fungus *Yarrowia lipolytica* via photosynthesis. In response, *Y. lipolytica* decomposes complex macromolecular organic matter into small molecules and CO_2_, which is beneficial for the growth of *C. pyrenoidesa* [6].

The active proteolytic peptides are composed of 3−20 amino acids; they are low-molecular weight bioactive molecules with relatively simple structures, exert minimal side effects, and can be directly absorbed by the body [7]. A study showed that pepsin hydrolysates of algae protein can improve glucose metabolism disorder and fatty liver caused by a high-fat diet [8]. Studies have shown that the active peptide (Val-Glu-Gly-Tyr) of the hydrolysates of alkaline protease can lower blood pressure by inhibiting the activity of angiotensin-converting enzyme (ACE) [9]. 

A free radical is a particle with unpaired electrons. Because of their high reactivity, free radicals can attack lipids and proteins of biological membranes, leading to cellular damage [10]. The antioxidant activity of proteolytic peptides depends on the number and type of amino acids and their position in the peptide chain. A mechanism has been proposed by which some peptides exhibit poor iron-chelation activity and can provide protons to quench free radicals [11]. Another hypothesis suggested that the peptides can act as chelators and effectively inhibit metallic and nonmetallic ion-catalyzed lipid peroxidation, as well as that catalyzed by hemoglobin, lipase, O^2−^, ·OH, and ROO· [12]. 

With the development of computer technology, studies of quantitative structure–activity relationships (QSARs) are improving and their scope of application is also expanding rapidly. The QSAR model is used to predict antimicrobial, antioxidant, and bitter peptides and ACE-inhibitory peptides from the amino acid sequence [13]. The antioxidant ability of a peptide is closely related to the type, quantity, and sequence of amino acids. The model established using the partial least squares method can predict the antioxidant activity of different peptide segments according to the amino acid composition of the peptide [14]. 

Recently, *C. pyrenoidesa* and its active substances have attracted attention as marine resources. The trypsin-hydrolyzed peptides (EHPs) of *C. pyrenoidesa* are a new type of natural antioxidant, which can be used in food, medicine, cosmetics, and for other purposes [15]. However, the mechanism by which EHPs perform antioxidative functions is unknown. This study was performed to (a) characterize the amino acid composition of EHPs obtained from *C. pyrenoidosa* in a co-culture system, (b) investigate the antioxidative activity of the EHP fraction and subsequently infer the mechanism involved in protecting human hepatoma HepG2 cells from 2,2’-azobis(2-amidinopropane) dihydrochloride (AAPH)-induced oxidative stress, and (c) characterize the structure–antioxidant activity relationship of EHPs in free radical systems using QSAR models, then verifying the relationships using human hepatoma HepG2 cells. This study provides useful information regarding the co-culture system of *C. pyrenoidosa* and *Y. lipolytica* (3:1 ratio), which has not been extensively studied. This study demonstrates EHPs with functional potential from a co-culture system of *C. pyrenoidosa* and *Y. lipolytica*.

## 2. Results and Discussion

### 2.1. Amino Acid Composition

The antioxidant properties of EHPs depend on the amino acid composition and properties of their side chain residues. In the present study, we detected 5.7% aromatic amino acids (AAAs), 22.4% negatively charged amino acids (NCAAs), and 48.1% hydrophobic amino acids (HAAs) in the EHPs (Table 1). In general, EHPs contain all essential amino acids and are rich in glutamate, which significantly affected the antioxidant properties [16].

### 2.2. EHP Sequence

High-performance liquid chromatography-mass spectrometry (HPLC-MS/MS) analysis was used to determine the amino acid sequence and molecular weight of EHP. The mechanism of trypsin action mainly includes identification of the target amino acids and cleavage of their C-terminal peptide bonds. The specificity of EHP has been widely verified and mainly hydrolyzes peptide chains composed of arginine or lysine carboxylic acids [17]. Thus, the restriction sites of trypsin in EHP include arginine and lysine. Studies have shown that the antioxidant activities of peptides are related to their molecular weight distribution. Peptides in the protein hydrolysate with high antioxidant activity were located in a lower molecular weight pool (<6500 Da) [15,18]. The results revealed 836 different peptides in EHP with molecular weights ranging from 639 to 3531 Da (Table S2). Among them, the molecular weight of EHP was mainly concentrated in the 1000−1500 Da range, followed by 1500–2000 Da (Table S3). The EHP predominantly contained certain low-molecular weight peptides that may act as antioxidants.

### 2.3. In Vitro Antioxidant Assay 

**Figure 1:**
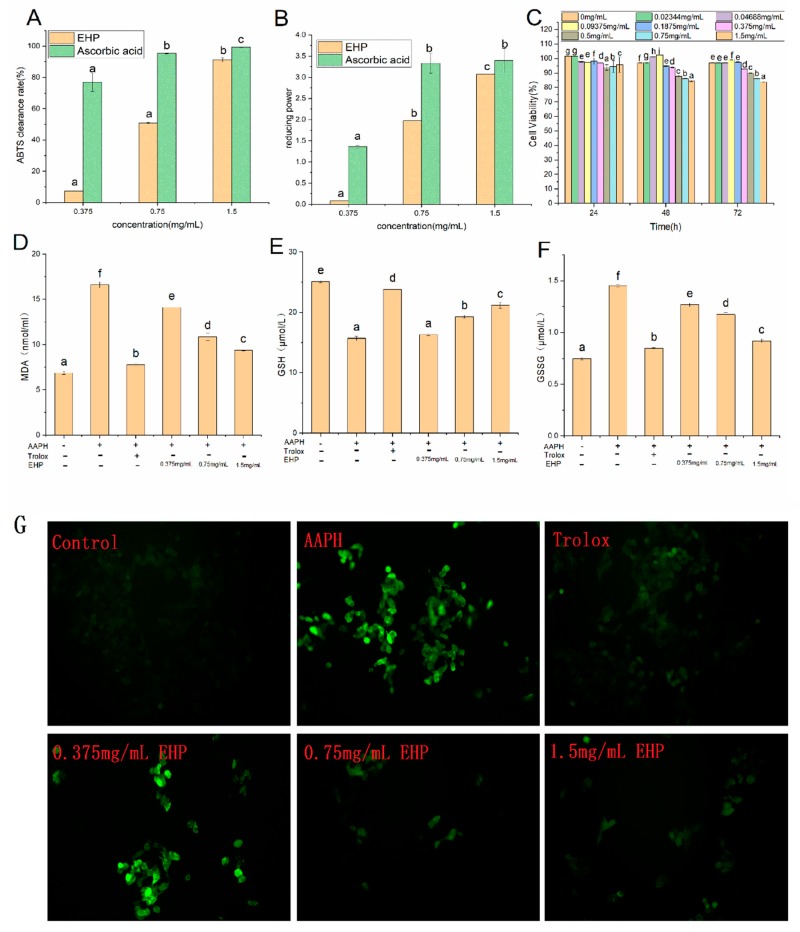
Antioxidative abilities of trypsin-hydrolyzed peptide (EHP) in vitro and in HepG2 cells. The 2,2-azobis(2-amidinopropane) dihydrochloride radical scavenging power of different concentrations of EHP (**A**). Reducing power of different concentrations of EHP (**B**). Effects of EHP on the viability of HepG2 cells (**C**). Effects of different concentrations of EHP on 2,2’-azobis(2-amidinopropane) dihydrochloride (AAPH)-induced changes in intracellular malondialdehyde (MDA) (**D**), glutathione (GSH) (**E**), and glutathione (GSSG) (**F**) levels in HepG2 cells. Data are the mean ± standard deviations of three independent experiments. Intracellular reactive oxygen species (ROS) scavenging capacities of blank group, AAPH group, Trolox, 0.375 mM EHP, 0.75 mM EHP, and 1.5 mM EHP at 180 min of incubation (**G**).

The 2,2’-azinobis-(3-ethylbenzthiazoline-6-sulfonate) (ABTS) radical scavenging test and reducing power test are traditional methods for evaluating the antioxidant activity of EHP [19]. Our results showed that EHP possessed reducing power and scavenging radical scavenging activity (Figure 1A,B). Furthermore, the reducing power and radical scavenging activity of EHP increased with EHP concentration (0.375−1.5 mg/mL). In particular, 1.5 mg/mL EHP showed the best reducing power and radical scavenging activity in those tested. In general, EHP contains more hydrophobic amino acids, which can increase the solubility of peptides in lipids and make it easier for the peptides to enter the target organ or tissue to better interact with radicals [20]. Aromatic and negatively charged amino acid contents were high, which can provide electrons that react with free radicals to reduce the number of free radicals and improve the antioxidant ability of EHP. Thus, the antioxidant properties of EHPs may depend on the amino acid composition.

### 2.4. Effect of EHP on Cell Viability

At present, HepG2 was widely used as a cell model for evaluating antioxidant activity, in which the nuclear factor erythroid 2-related factor 2 (Nrf2) pathway is an important regulator of oxidative stress [21,22]. HepG2 developed an adaptive cytoprotective response by up-regulating antioxidant defense systems including the Nrf2-ARE signaling pathway [23]. The effect of EHP on cell viability was evaluated using the cell counting kit-8 assay. As shown in Figure 1C, cell viability decreased in a time- and EHP dose-dependent manner. Cell viability decreased with increasing treatment time, indicating damage in HepG2 cells. However, no significant differences were observed in cell viability at values higher than 80% after 48 h, indicating that inhibition had reached a plateau. Thus, we used the 48 h time point in subsequent follow-up experiments. In terms of peptide concentration, cell viability decreased with increasing treatment concentrations, indicating that EHP had negligible cytotoxicity towards HepG2 cells at low concentrations. Therefore, the EHP concentrations were set to 0.375, 0.75, and 1.5 mg/mL for further analysis of the protective effects of different concentrations of EHP on HepG2 cells.

### 2.5. Effects of EHP on Reactive Oxygen Species (ROS) Generation

High levels of ROS cause lipid peroxidation, resulting in membrane damage and protein degradation, leading to apoptosis [24]. Therefore, ROS levels often reflect the degree of oxidative damage in HepG2 cells. As shown in Figure 1G, AAPH treatment increased the fluorescence intensity compared to that of the positive control, indicating oxidative stress injury in the cells. In the AAPH group, the fluorescence intensity of the samples decreased significantly (*p* < 0.05) with increasing EHP concentration. The ROS level decreased when the cells were treated with high concentrations (1.5 mg/mL) of EHP, indicating that EHP can reduce ROS levels. The EC_50_ value of Trolox, a positive control, and EHP were 0.08 ± 0.002 and 0.28 ± 0.01 mg/mL, respectively. A study showed that the EC_50_ value of alcohol-soluble peptide from corn bran was 2.85 mg/mL, which was significantly higher than that of EHP [25]. Therefore, EHP showed antioxidant properties. 

### 2.6. Effect of EHP on Malondialdehyde (MDA) Levels

Free radicals act on lipids to form peroxide, and the end-product of oxidation is MDA. Therefore, the amount of MDA often reflects the extent of cellular oxidative stress injury [26]. As shown in Figure 1D, the AAPH group generated more MDA than the EHP and Trolox groups. Trolox was used as a positive control, as it is a good antioxidant that can be used in the HepG2 model. In the sample group, the degradation of cell membranes was inversely proportional to the EHP concentration. Therefore, after pretreatment with different concentrations of EHP, the level of AAPH-induced lipid peroxidation in these cells was obviously lower than those in the negative control group, and the concentration of MDA decreased with increasing EHP concentrations. Thus, 1.5 mg/mL EHP may significantly (*p* < 0.05) reduce oxidative damage in HepG2 cells.

### 2.7. Effect of EHP on Reduced Glutathione (GSH) and Oxidized Glutathione (GSSG) Levels 

GSH is the main non-enzymatic antioxidant involved in cellular defense against oxidative stress. It can protect against ROS, free radicals, and electrophilic metabolites; additionally, it can conjugate with and detoxify several types of toxic and carcinogenic compounds [27]. Therefore, the consumption of GSH and generation of GSSG reflect the degree of oxidative damage in HepG2 cells. As shown in Figure 1E,F, the content of GSH was lowest in the AAPH group, whereas that of GSSG was the highest, indicating that AAPH can cause oxidative damage in HepG2 cells. The results obtained in the blank group were opposite those observed in the AAPH group. Compared to the levels of GSH and GSSG in the AAPH group, those in the EHP groups were increased significantly (*p* < 0.05) to 0.6−5.5 μmol/L and decreased to 0.2−0.6 μmol/L, respectively. Significant differences (*p* < 0.05) were observed between the Trolox group (23.8 ± 0.24 μmol/L) and high-level treatment groups (21.16 ± 0.49 μmol/L), indicating that the high level of EHP antioxidant activity was similar to that of Trolox. Thus, the high concentration of EHP possessed marked antioxidant activity via the protective effect of reduced GSH.

### 2.8. Effect of EHP on Superoxide Dismutase (SOD), Catalase (CAT), and Glutathione Peroxidase (GSH-PX) Activities

**Figure 2:**
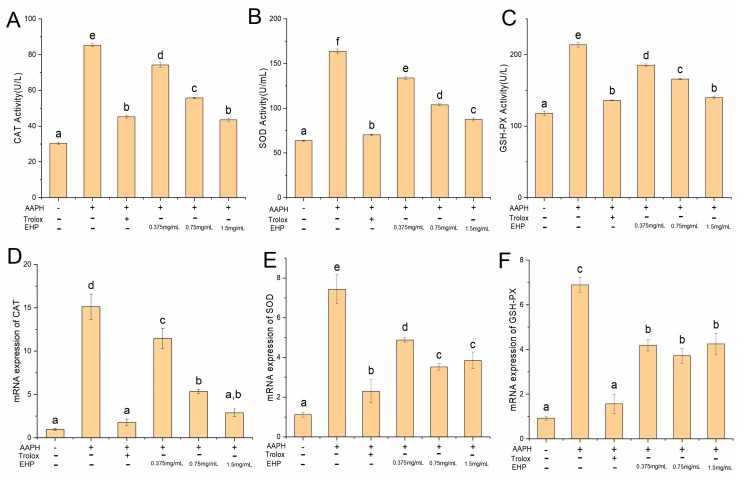
Effect of EHP on catalase (CAT), superoxide dismutase (SOD), and glutathione peroxidase (GSH-PX) activities and mRNA expression. Effects of EHP on AAPH-induced changes in cellular antioxidant enzyme levels in HepG2 cells. CAT (**A**), SOD (**B**), and GSH-Px (**C**). The mRNA levels of AAPH-induced oxidative stress metabolism-related genes, including CAT (**D**), SOD (**E**), and GSH-PX (**F**), in HepG2 cells pretreated with different concentrations of EHP. Each mRNA level was normalized to those of GAPDH and expressed relative to the control. Data are the mean ± standard deviations of three independent experiments.

As shown in Figure 2A–C, the CAT, SOD, and GSH-PX activities in the AAPH group were significantly (*p* < 0.05) higher than those in the blank and positive control groups. However, compared to the AAPH group, CAT activity decreased significantly to 11.07, 29.43, and 41.7 U/mL; SOD activity decreased significantly (*p* < 0.05) to 29.66, 59.8, and 76.16 U/mL; and GSH-PX activity decreased significantly (*p* < 0.05) to 28.63, 47.93, and 74.06 U/mL, respectively, with gradual increases in the EHP concentration (0.375, 0.75, and 1.5 mg/mL). Some studies showed that Trolox is a potent antioxidant that releases active hydrogen on the hydroxyl group and then captures the free radical, thereby blocking the free radical chain reaction [28]. This agrees with the CAT, SOD, and GSH-PX activities observed in the Trolox group, which were 45.26 ± 0.85, 70.4 ± 0.8, and 136.1 ± 0.57 U/mL, respectively. These results indicate that EHP protects the activities of CAT, GSH-Px, and SOD from AAPH-induced changes. In general, the EHP contains more hydrophobic amino acids, which can increase the solubility of peptides in lipids and make it easier for the peptides to enter the target organ or tissue to better interact with radicals. Aromatic and negatively charged amino acid contents were high, which can provide electrons that react with free radicals to reduce the number of free radicals and improve the antioxidant ability of EHP. Thus, the antioxidant mechanism of EHP may involve binding to protons and blocking of free radical chain reactions that may depend on the amino acid composition.

### 2.9. mRNA and Protein Expression of Nrf2, Kelch-Like ECH-Associated Protein-1 (Keap1), CAT, SOD, and GSH-PX

Under unstressed conditions, nuclear factor erythroid 2-related factor 2 (Nrf2) associates with Kelch-like ECH-associated protein-1 (Keap1) via the ubiquitin-proteasome pathway [29]. In the presence of oxidative stress, the cysteine residues in Keap1 form disulfide bonds, which release Nrf2; thus, Nrf2 accumulates within the cell and induces the expression of antioxidant and detoxification enzymes, including CAT, SOD, and GSH-PX [30]. 

**Figure 3:**
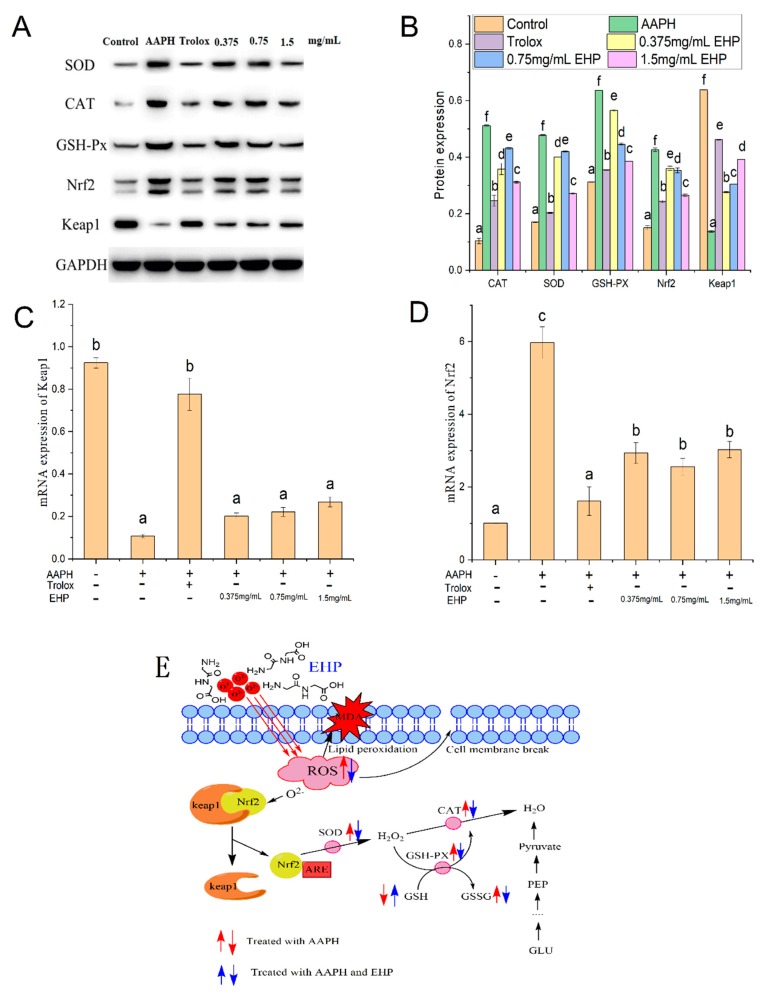
Antioxidant mechanism. The mRNA expression levels of antioxidant signaling pathway-related genes, including encoding nuclear factor erythroid 2-related factor 2 (Nrf2) (**A**) and Kelch-like ECH-associated protein-1 (Keap1) (**B**) in HepG2 cells pretreated with different concentrations of EHP. Each mRNA level was normalized to that of GAPDH and expressed relative to the control. Hepatic expression of oxidative stress metabolism-related proteins and antioxidant signaling pathway-related proteins (**C**,**D**) in HepG2 cells pretreated with different concentrations of EHP. Each protein expression level was normalized to that of GAPDH and expressed relative to the control and positive control levels. A possible hypothesis to explain these effects is that AAPH-induced oxidative stress increased reactive oxygen species (ROS) generation and caused Keap1 oxidation, which released Nrf2, thereby activating the antioxidant enzymes. EHP can provide electrons, which allows it to directly quench free radicals and act as an antioxidative (**E**). Data are the mean ± standard deviations of three independent experiments.

The mRNA (Figure 2D–E) and protein levels (Figure 3A–B) of CAT, SOD, and GSH-PX in HepG2 cells treated with 100 μM AAPH were increased significantly (*p* < 0.05) compared to in the control group, indicating that 100 μM AAPH caused oxidative stress to the cells, although the extent of cellular damage was not significant. This is consistent with the results reported in previous studies [31,32]. We observed that the *Keap1* gene and protein expression levels in the AAPH group were 11% and 20% lower than those in the control group, respectively (Figure 3A–C). Furthermore, the Nrf2 gene and protein levels in the AAPH group were 20% and 17% higher than those in the control group, respectively (Figure 3A,B,D). This was mainly because of changes in the structure of Keap1 after AAPH treatment, which led to the release of Nrf2. 

However, compared to the AAPH group, the mRNA and protein levels of CAT, SOD, and GSH-PX in HepG2 cells treated with different concentrations of EHP or Trolox were decreased significantly (*p* < 0.05). Particularly, the mRNA and protein levels of CAT, SOD, and GSH-PX decreased after treatment with high concentrations EHP or Trolox. In contrast, the mRNA and protein levels of Nrf2 in the sample and Trolox group were lower than those in the AAPH group. This was mainly because Nrf2 forms a complex with Keap1 in the cytoplasm and is rapidly degraded by the ubiquitin-proteasome system under normal physiological conditions to ensure that the normal redox system in the body was in equilibrium [33]. Therefore, EHP contains amino acids that may provide electrons, enabling it to directly quench free radicals and act as an antioxidant (Figure 3E).

### 2.10. QSAR Model Establishment and Verification

**Figure 4:**
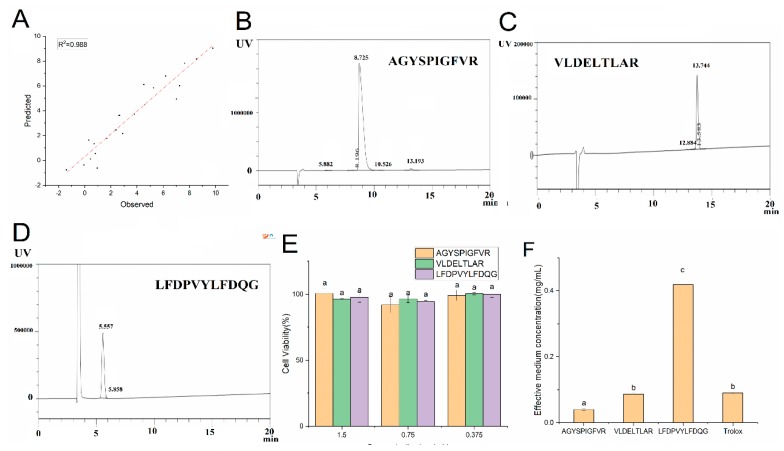
Quantitative structure–activity relationships (QSAR) model: Plot showing the comparison of observed and predicted linear relationship in the QSAR model (**A**). Prediction of antioxidant model of peptide sequence using the QSAR model. Three peptides were synthesized to verify the accuracy of the model, including AGYSPIGFVR (**B**), VLDELTLAR (**C**), and LFDPVYLFDQG (**D**). Effects of the three peptides on the viability of HepG2 cells (**E**), and the concentration required for 50% of the maximal AAPH-induced oxidative stress in HepG2 cells (**F**). Data are the mean ± standard deviations of three independent experiments.

To investigate the relationship between the peptide sequence and antioxidant activity, we randomly divided the whole dataset into a training set and test set, and then validated the training set model with the test set. The cumulative multiple correlation coefficient for the calibration set (*R*^2^) was 0.999 (Figure 4A). The cumulative cross-validation coefficient for the calibration set (*Q*^2^) was 0.587 and root mean square error for the prediction dataset was 1.54. This shows that the QSAR model had good external prediction ability. We used this model to predict all sequences of EHP from the co-culture system (Table S4). The peptide sequence AGYSPIGFVR showed the highest predicted antioxidant activity, followed by the peptide sequence VLDELTLAR with an intermediate predicted antioxidant activity, and the peptide sequence LFDPVYLFDQG showed the lowest predicted antioxidant activity. Therefore, we synthesized three typical peptides to verify their antioxidative effects. HPLC analysis revealed an easily detected single symmetrical target peak; the impurities were limited in number and amount, indicating the purity of AGYSPIGFVR (Figure 4B). The VLDELTLAR and LFDPVYLFDQG peptides (Figure 4C,D) were 98.77% and 99.58% pure, respectively.

The effect of the peptides on cell viability was evaluated using the cell counting kit-8 (CCK-8) assay. As shown in Figure 4E, there were no significant differences in cell viability (>80%) after peptide treatment, indicating that the peptides did not damage the HepG2 cells. As shown in Figure 4F, the EC_50_ value of Trolox and VLDELTLAR for the cell viability in AAPH-induced HepG2 cells were 0.08 ± 0.002 and 0.09 ± 0.001 mg/mL, respectively. AGYSPIGFVR showed the highest antioxidant activity with an EC_50_ value of 0.04 ± 0.002 mg/mL, which was significantly (*p* < 0.05) lower than that of Trolox. However, the EC_50_ value of LFDPVYLFDQG was higher than that of Trolox. We observed that the antioxidant activities of peptides were in the order of AGYSPIGFVR > VLDELTLAR > LFDPVYLFDQG. Thus, the QSAR model can predict the antioxidant activity of peptides.

The presence of hydrophobic amino acids was effective against the peroxyl radical [34]. AAAs and NCAAs have been shown to enhance the antioxidant potential of EHP via proton-donation ability, electron-donation ability, and direct lipid radical scavenging [35]. Several studies have shown that the reducing power and radical scavenging activity can be affected by the presence of EHP or amino acids with electron donating capacity [36]. Therefore, amino acid composition may be a favorable factor in the antioxidant activities of EHP.

Previous studies showed that pretreatment with EHP-reduced AAPH-induced ROS generation in HepG2 cells [21]. Previous studies have shown that GSSG and H_2_O are generated in the reaction of GSH with H_2_O_2_ when AAPH free radicals attack HepG2 cells [37]. HepG2 cells contain various antioxidative enzymes, including CAT, SOD, and GSH-PX, which protect against oxidative stress. H_2_O_2_ is converted to H_2_O via CAT or GSH-PX catalysis [38]. SOD is an important free radical scavenger in all organisms [39]. However, Trolox possesses good antioxidant capacity, mainly because of its adsorption and redox capacity in experiments [28]. Thus, EHP contains HAAs, AAAs, and NCAAs may contribute to peroxidation inhibition and electron donating capacity, thereby facilitating interactions with radical species to directly scavenge them.

Previous studies showed that Nrf2 can regulate the expression of antioxidant enzymes, thereby reducing the level of cell-damaging free radicals [40]. A previous study showed that Trolox provides electrons to free radicals to interrupt chain reactions, thereby acting as an antioxidant [28]. Therefore, EHPs appear to contain amino acids that can provide electrons, enabling direct quenching of free radicals and function as an antioxidant.

The corresponding criteria for a QSAR model to obtain high predictive power were as follows: R > 0.6; Q > 0.5 [41]. The EC_50_ value of Trolox was similar to that obtained previously [25]. Therefore, AGYSPIGFVR showed the highest antioxidant activity in EHPs. 

## 3. Materials and Methods 

### 3.1. Materials

*Chlorella pyrenoidesa* and *Y. lipolytica* were obtained from the Laboratory of Food Science and Engineering, South China University of Technology. Yeast mold (YM) medium was obtained from Qingdao Hope Bio-Technology Co., Ltd. (Shandong, China). Other reagents used were of analytical grade.

### 3.2. EHP Preparation from Symbiotic Culture

*Chlorella pyrenoidesa* was activated with sterile Tris-acetate phosphate medium and *Y. lipolytica* with sterile YM medium at 25 °C with shaking at 150 rpm in the presence of light for 3 and 2 days, respectively. The two co-culture groups consisted of 15 mL active *C. pyrenoidesa* resuspended in sterile 2 L Tris-acetate phosphate medium, which was also mixed with 2 mL active *Y. lipolytica*. The symbiotic system was incubated for 5 days at 25 °C in the presence of light with shaking at 150 rpm. After collection and centrifugation of the cells at 1200× *g* for 15 min, the supernatant was removed, and the pellet was washed twice with sterile water. Protein was extracted from the cultures as previously described with some modifications [42]. First, 1 g *C. pyrenoidesa* was dissolved in 20 mL distilled water, followed by slow addition of 0.8 g sodium hydroxide at 47 °C in an ultrasonic bath. The pH was adjusted to 7.0 with HCl. The solution was centrifuged at 1200× *g* for 20 min and the supernatant was cooled to 4 °C. Subsequently, a fourfold volume of 95% cold ethanol was added to the supernatant and incubated for 5 h. This was followed by centrifugation at 1200 × *g* for 20 min to collect the precipitate. The proteins were dissolved in 0.1 mol/L phosphate-buffered saline, adjusted to pH 6.5 and 37 °C. Next, the proteins were hydrolyzed with trypsin for 5 h. The liquid was placed in boiling water for enzyme inactivation and centrifuged at 1200× *g* for 10 min to collect the supernatant. Finally, the samples were vacuum freeze-dried at −40 °C to obtain the EHP powder.

### 3.3. Qualitative Proteomics Assay Using Liquid Chromatography (LC)-MS/MS 

The protein powder was dissolved in 200 μL Buffer 1 (8 M urea and 0.1 M Tris-HCl, pH 8.5) and placed in a 10 kDa ultrafiltration tube and centrifuged at 12,000× *g* for 10 min at 4 °C. This was repeated until the entire solution had passed through the filter. Next, 200 μL Buffer 2 (8 M urea and 0.1 M Tris-HCl, pH 8.0) was added to each ultrafiltration tube and centrifuged twice at 12,000× *g* for 10 min at 4°C, after which 200 μL 25 mM (NH_4_)_2_CO_3_ solution was added to each ultrafiltration tube and centrifuged under the same conditions. This was followed by addition of 100 μL of 0.01 μg/μL trypsin solution and incubation in a 37 °C water bath for 14 h. Subsequently, the sample was centrifuged at 12,000× *g* for 10 min, followed by addition of 100 μL 25 mM (NH_4_)_2_CO_3_ solution and centrifugation under the same conditions. The filtered liquor was dried by rotating vacuum centrifugation to obtain EHP powder, which was used for mass spectrometric analysis [43].

Prior to LC-MS/MS analysis, the EHP powder was dissolved in mixed liquor consisting of 0.1% formic acid and 2% acetonitrile and then desalted in a Chrom XP C_18_ column (3 μm, 120 A) for 5 min at a flow rate of 4 μL/min. The EHP was separated on a C_18_ column (75 μm × 150 mm, 3 μm, 120 A) (Chrom XP Eksigent, Redwood City, CA, USA) using a Ekspert nano LC (SCIEX, Concord, Ontario, Canada) coupled to a Triple TOF 6600 system (SCIEX) and nanoliter spray III ion source (SCIEX). The experimental conditions were as follows: spray voltage of 2.4 kV, air curtain voltage of 35 PSI, atomizing air pressure of 12 PSI, and heating temperature of 150 °C. The gradient was 8% mixed liquor of 95% acetonitrile in 0.1% formic acid for 60 min, which was increased to 38% mixed liquor of 95% acetonitrile in 0.1% formic acid for 30 min. 

The spectra were acquired using the software PEAKS Studio 8.5 (version 8.5, Bioinformatics Solutions, Inc., Waterloo, Canada) and searched against the *C. pyrenoidesa* protein database, which contained peptides derived from trypsin hydrolysis, with a parent ion mass error tolerance of 15 ppm and fragment ion mass error tolerance of 0.05 Da. The false-positive rate of EHP was controlled at a 0.5% false positive discovery rate [44].

### 3.4. Amino Acid Analysis

The amino acid composition of the co-culture system-derived EHP was determined using an A300 auto amino acid analyzer (Membra Pure, Bodenheim, Germany) according to our previous study with minor modifications [45]. The EHP was first treated with 6 M HCl at 110 °C for 24 h and then derivatized with ninhydrin to measure the total amino acid composition. The free amino acid composition was measured after the EHP was mixed with 15% sulfosalicylic acid and incubated for 1 h, followed by centrifugation at 1200× *g* at 4 °C for 10 min to collect the liquid supernatant. Appropriate dilutions of the obtained supernatant were filtered through a 0.22 μm filter membrane before total and free amino acid analysis. The amino acid standards were used as external standards.

### 3.5. In Vitro Evaluation of Free Radical Scavenging Capabilities 

The 2,2’-azino-*bis* (3-ethylbenzothiazoline-6-sulfonic acid) (Sigma-Aldrich, St. Louis, MO, USA) free radical scavenging power of EHP was measured as described by Floegel et al. [46]. Briefly, 5 mL 7 mM 2,2’-azinobis-(3-ethylbenzthiazoline-6-sulfonate) (ABTS) was mixed with 5 mL 2.45 mM 2,2-azobis(2-amidinopropane) dihydrochloride. The mixture was incubated in the dark for 12 h. The blue-green ABTS^+^ solution was diluted with fresh phosphate-buffered saline (PBS) until the absorbance was 0.70 ± 0.02. Next, 0.08 mL of vitamin C standard or EHP (1.5, 0.75, and 0.375 mg/mL) was mixed with 0.6 mL ABTS+ solution and incubated for 30 min in the dark; absorbance was recorded at 732 nm (As). In the control group, 0.08 mL vitamin C standard or EHP (1.5, 0.75, and 0.375 mg/mL) was mixed with 0.6 mL PBS solution (Ac); PBS was used rather than ABTS^+^ solution in the blank group (Ab). The scavenging capacity of ABTS free radicals was then determined using the following formula: ABTS scavenging activity (%) = 1 − [(As − Ac)/Ab] × 100.

The reducing power of EHP was measured as described by Xie et al. [47]. Briefly, 1 mL EHP (1.5, 0.75, and 0.375 mg/mL) was added to a solution containing 2.5 mL 0.2 M phosphate buffer (pH 6.6) and 2.5 mL 1 g/100 mL potassium ferricyanide, and incubated at 50 °C for 20 min. Subsequently, 2.5 mL trichloroacetic acid solution (10 g/100 mL) was added to stop the reaction. After centrifugation at 1650× *g* for 10 min, 2.5 mL of the upper layer was diluted with 2.5 mL deionized water and 0.5 mL of 0.1% ferric chloride. After a 10 min reaction, the absorbance was recorded at 700 nm. The blank group consisted of the solvent rather than the sample, and vitamin C (1.5, 0.75, and 0.375 mg/mL) was used as the positive control. 

### 3.6. Determination of Antioxidative Abilities of EHP in HepG2

#### 3.6.1. Cell Culture 

Cell culture was performed as previously described with some modifications [48]. The HepG2 cells were cultured in sterile Dulbecco’s modified Eagle’s medium (DMEM) with 10% fetal calf serum at 37 °C in a 5% CO_2_ incubator. This was followed by centrifugation at 1200× *g* for 20 min to collect the precipitate, which was washed three times with PBS. 

#### 3.6.2. Cell Viability Assay

After culturing at 37 °C in a 5% CO_2_ incubator for 24 h, the HepG2 cells were resuspended in sterile DMEM to adjust the cell concentration to 1 × 10^5^ cells/mL. Next, the HepG2 cells were mixed with 100 μL EHP of different concentrations (0.02344, 0.4688, 0.09375, 0.1875, 0.375, 0.5, 0.75, and 1.5 mg/mL) for 24, 48, or 72 h, followed by addition of cell counting kit-8 (CCK-8) reagent; the absorbance was recorded at 450 nm (A_s_). In the control group (A_c_), HepG2 cells were mixed with 100 μL DMEM and incubated for 24, 48, or 72 h, followed by addition of CCK-8 reagent and absorbance was measured at 450 nm (As). The blank control group (A_b_) lacked HepG2 cells, EHP, and CCK-8 reagent [32]. Cell viability was calculated using the formula:

Cell viability (%) = [(A_s_ − A_b_)/(A_c_ − A_b_)] × 100.

#### 3.6.3. Measurement of ROS Production 

Intracellular ROS was quantified as previously described with some modifications [49]. HepG2 cells were cultured in 2,7-dichlorodi-hydroflurescein diacetate (DCFH-DA) as follows: 1 μL DCFH-DA was first diluted in 1000 μL serum-free medium, in which the cells were incubated for 30 min at 37 °C in a 5% CO_2_ incubator. Serum-free medium was used to wash the cells three times. Next, 1.5, 0.75, and 0.375 mg/mL EHP or 1.5 mg/mL Trolox in serum-free DMEM medium were added to the respective sample groups. An equal volume of DMEM was added to the control group and AAPH group and cultured at 37 °C in a 5% CO_2_ incubator for 20 min; the cells were then centrifuged at 12,000× *g* for 10 min and washed three times with PBS. The sample and AAPH groups were treated with 200 μmol/L AAPH, whereas the control group was treated with sterile water and cultured under the same conditions. Changes in fluorescence intensity were observed under an inverted fluorescent microscope (DMIL LED, Leica, Wetzlar, Germany).

#### 3.6.4. Determination of MDA, GSH, and GSSG Levels

Six hundred microliters of HepG2 cells were inoculated into 96-well plates at the density of 1 × 10^5^ cells/mL. The samples were treated with 1.5, 0.75, or 0.375 mg/mL EHP or 1.5 mg/mL Trolox, whereas the control group was treated with an equal volume of serum-free medium; all samples were cultured for 3 h and then washed three times with PBS. This was followed by addition of 200 μmol/L AAPH and culture at 37 °C in a 5% CO_2_ incubator for 3 h; the cells were then centrifuged at 12,000× *g* for 10 min and washed three times with PBS. The cells were added to double ultrapure water and incubated in an ice-water bath for 10 min and centrifuged (1200× *g*, 10 min, 4 °C) to obtain the cell lysis buffer. The levels of MDA, GSH, and GSSG were determined using the bicinchoninic acid method and kits (Beyotime Institute of Biotechnology, Shanghai, China).

#### 3.6.5. Determination of SOD, CAT, and GSH-PX Levels

Six hundred microliters of active HepG2 cell were inoculated into 96-well plates at a density of 1 × 10^5^ cells/mL. The sample groups were treated with 1.5, 0.75, or 0.375 mg/mL EHP or 1.5 mg/mL Trolox. The control group was treated with an equal volume of serum-free medium. All samples were cultured for 3 h, after which the cells were washed three times with PBS. This was followed by addition of 200 μmol/L AAPH and culture at 37 °C in a 5% CO_2_ incubator for 3 h. The media was discarded, and the cells were washed three times with PBS (pH 7.4), after which they were added to double ultrapure water in an ice-water bath and incubated for 10 min. Next, the cells were centrifuged (1200× *g*, 10 min, 4 °C) to obtain the cell lysis buffer. The levels of the SOD, CAT, and GSH-Px were determined using the bicinchoninic acid method and kits for SOD, CAT, and GSH-PX (Beyotime Institute of Biotechnology).

#### 3.6.6. Analysis of mRNA Expression of SOD, CAT, GSH-PX, Keap1, and Nrf2 

The total RNA from HepG2 cells was extracted using Trizol (Ambion, Cambridgeshire, UK) according to the manufacturer’s instructions. DNA was removed from <1 ng total RNA using 1 μL 3 U/μL DNase I in 1 μL 10 × DNase I buffer and 10 μL diethyl pyrocarbonate -treated H_2_O at 37 °C for 30 min; the reaction was stopped by adding 1 μL ethylenediaminetetraacetic acid and incubating at 65 °C for 10 min. The reaction liquid was mixed with the same amount of phenol:chloroform:isoamyl alcohol (25:24:1), centrifuged at 1200× *g* for 5 min to collect the supernatant, mixed with 3 M sodium acetate trihydrate and cold anhydrous ethanol, and incubated at −20 °C for 60 min. The samples were centrifuged at 1200× *g* for 5 min to collect the precipitate, which was resuspended in DEPC-H_2_O. The reverse transcription reaction contained 500 ng total RNA, 4 μL 5 × reaction buffer, 1 μL oligo (dT)18 primer, 2 μL 10 mM deoxy-ribonucleoside triphosphate mix, 1 μL RiboLock RNase inhibitor (20 U/μL),1 μL RevertAid M-MuLV reverse Transcriptase (200 U/μL), and 20 μL double distilled (DNase-free). The reaction was incubated at 42 °C for 60 min, and then at 70 °C for 15 min. Reverse transcription quantitative polymerase chain reaction (RT-qPCR) was performed using a SYBR Green PCR kit (KM4101, KAPA Biosystems, Wilmington, DE, USA) according to the manufacturer’s instructions. The RT-qPCR conditions were as follows: 1 cycle of 95 °C for 10 min, and 40 cycles of 95 °C for 10 s and 60 °C for 1 min. All samples were assayed in triplicate and the data were analyzed using the ΔΔCT method [50]. GAPDH primers were added as the internal control. The primers used in this assay are listed in Table S1.

#### 3.6.7. Analysis of Protein Levels of SOD, CAT, GSH-PX, Keap1, and Nrf2 

Protease and phosphatase inhibitors were added to the HepG2 cells, which were split at 4 °C, heated for 10 min, and centrifuged at 1200× *g* for 5 min to collect the supernatant. Next, the supernatants were boiled for 10 min and centrifuged at 1200× *g* for 5 min, followed by electrophoresis on 10% sodium dodecyl sulfate polyacrylamide gel. The proteins were transferred onto a polyvinylidene fluoride membrane (Millipore, Billerica, MA, USA) and blocked with 5% skim milk for 2 h at room temperature. The membranes were incubated with diluted antibodies (SOD, CAT, GSH-Px, Nrf2, Keap1, and GAPDH, respectively) for 1 h. After washing five times with PBS-Tween 20 (PBST), the membranes were incubated with horseradish peroxidase-conjugated secondary antibodies at 1:10,000 dilutions for 1 h, and then washed three times in PBST. Subsequently, the proteins were detected using an enhanced chemiluminescence (ECL) kit (Millipore). GAPDH was used as the loading control [50].

### 3.7. Quantitative Structure–Activity Relationship Modeling

The oxygen radical absorbance capacity database, which contains 23 samples from different hydrolysates, is shown in Table 2 [51]. The electronic properties amino acid descriptor was used to describe the peptides of the dataset or sample using the two-terminal position numbering method [41]. For QSAR modeling, the databases were randomly divided into a calibration set and prediction set in a ratio of 2:1. The calibration set was used to build the QSAR model and perform internal validation, and the prediction set was used for external validation. The internal validation and external validation systems were designed to evaluate the quality and predictive ability of the model in the partial least squares program. Several evaluation functions were used to evaluate the predictive ability of the QSAR model.

### 3.8. Validation Experiments

On the basis of the QSAR model test results, three peptides with strong, moderate, and weak antioxidant activities were selected for validation experiments. The peptides were synthesized by Shanghai Science Peptide Biological Technology Co., Ltd. (Shanghai, China). Next, the effect of the peptides and Trolox (0.375, 0.75, and 1.5 mg/mL) on the viability of HepG2 cells was investigated. The EC_50_ values of the three synthetic peptides were determined.

### 3.9. Median Effective Concentration (EC_50_) Assay 

The HepG2 cells were cultured in DCFH-DA as follows: 1 μL DCFH-DA was diluted in 1000 μL serum-free medium and incubated for 30 min at 37 °C in a 5% CO_2_ incubator with the cells. Serum-free medium was used to wash the cells three times. The sample groups were treated with 1.5, 0.75, and 0.375 mg/mL peptide in serum-free DMEM. The control and AAPH groups were treated with equal volumes of DMEM and cultured at 37 °C in a 5% CO_2_ incubator for 20 min, after which they were centrifuged at 12,000× *g* for 10 min and washed three times with PBS. Similar to the sample groups, the AAPH group was treated with 200 μmol/L AAPH and the control group with sterile water; both groups were cultured under the same conditions. Fluorescence intensity was measured at excitation and emission wavelengths of 488 and 525 nm, respectively, at every 10 min within 1 h (TECAN, Maennedorf, Switzerland). The same method was used for treatment with Trolox as the positive control. The HepG2 cells were treated with different concentrations of peptides and Trolox, and the fluorescence attenuation curve was obtained. The CAA values were calculated according to the integrated area under the fluorescence versus time curve generated for each sample and the standard (Trolox) according to the following equation:

CAA unit = 100 − (∫SA/∫CA) × 100

where ∫SA and ∫CA are the integrated areas under the fluorescence versus time curves generated from the sample and control, respectively. After calculating the integral area, the median effective concentration (EC_50_) was determined from the plot of log (CAA unit/(100 − CAA unit)) versus log (dose) [49].

### 3.10. Statistical Analysis

Data are expressed as the mean ± standard deviation (SD) of three replicates. Significant differences between the means of parameters were calculated using Duncan’s multiple-range test of the SPSS 17.0 software (SPSS, Inc., Chicago, IL, USA). *P*-values less than 0.05 were considered to indicate statistical significance. 

## 4. Conclusions

Active peptides have attracted increasing attention for the production of potential bioactive components with high value. In this study, EHPs were extracted from a 5 day old co-culture of *C. pyrenoidosa* with *Y. lipolytica* (3:1 ratio). The EHPs contained 836 different amino acid sequences with molecular weights ranging from 639 to 3531 Da. The sample consisted of various amino acids with antioxidant abilities, including NCAAs, AAAs, and HAAs, which possessed free radical-scavenging capabilities and protected against AAPH-induced oxidative stress in HepG2 cells by suppressing ROS generation and preventing MDA formation. However, the mRNA and protein expression levels and activities of antioxidation-associated enzymes were significantly lower in the treated group than in the control group (without EHP). The antioxidant activity of 1.5 mg/mL EHP was equivalent to that of Trolox. We predicted that the electrons were released via the antioxidant mechanism of EHP action, possibly enabling EHP to directly quench free radicals and act as an antioxidant. Therefore, the EHPs scavenged free radicals directly outside the cell, without an Nrf2/ARE-mediated increase in enzyme activity. The QSAR model was established to identify EHP sequences that may possess antioxidant activity. The results revealed the accuracy of the model for predicting EHP antioxidant activity. These findings highlight a rapid method for identifying antioxidant peptides and provide useful information regarding bioproducts in the area of algal and yeast research. Although we verified the antioxidant activity of EHPs in vitro and at the cellular level, the precise mechanism of the protective effect of EHPs on cells under oxidative stress in vivo remains unclear. Further studies are needed to evaluate the antioxidant activity of EHPs in animal models. Our co-culture system may provide useful information for bioproducts in the area of algal and yeast research.

## Figures and Tables

**Table 1:** Total amino acid and free amino acid composition of the peptides.

Amino Acid	Total (nmol/mg)	Free (nmol/mg)
Asp	17.02 ± 1.38 ^ab^	ND ^c^
Thr	8.10 ± 0.02 ^a^	ND ^c^
Ser	6.40 ± 0.1 ^a^	ND ^c^
Glu	18.71 ± 1.84 ^b^	0.038 ± 0.02 ^b^
Gly	13.42 ± 1.27 ^a^	0.29 ± 0.09 ^a^
Ala	22.20 ± 1.31 ^a^	ND ^c^
Cys	ND ^c^	ND ^c^
Val	14.55 ± 0.41 ^a^	ND ^c^
Met	ND ^c^	ND ^c^
Ile	11.03 ± 1.12 ^b^	0.21 ± 0.01 ^a^
Leu	15.00 ± 1.13 ^a^	0.69 ± 0.02 ^a^
Tyr	6.21 ± 0.22 ^a^	0.29 ± 0.01 ^a^
Phe	2.97 ± 0.59 ^b^	0.40 ± 0.01 ^a^
His	0.26 ± 0.01 ^a^	ND ^c^
Lys	10.06 ± 0.41 ^b^	0.45 ± 0.04 ^b^
Arg	8.80 ± 1.64 ^b^	0.60 ± 0.02 ^a^
Pro	4.91 ± 0.3 ^a^	ND ^c^
HAAs^d^	76.87 ± 5.08 ^a^	1.59 ± 0.05 ^a^
NCAAs^e^	35.73 ± 3.22 ^b^	0.038 ± 0.02 ^a^
AAAs^f^	9.18 ± 0.81 ^a^	0.69 ± 0.02 ^a^
Sum	159.64 ± 11.75 ^b^	2.96 ± 0.24 ^a^

^a^ Mean ± standard deviation (*n* = 3). ^b^ Different letters (a,b) in the same column indicate significant difference between amino acids (*p* < 0.05). ^c^ The amino acid was undetectable in this study. ^d^ Hydrophobic amino acids (HAAs), including Ala, Val, Ile, Leu, Tyr, Phe, Pro, Met, and Cys; ^e^ negatively charged amino acids (NCAAs), including Asp and Glu; ^f^ aromatic amino acids (AAAs), including Phe and Tyr.

**Table 2:** QSAR database.

No	Sequence	Activity
1	WY	7.67
2	WYS	4.45
3	WYSL	4.52
4	WNIP	15.47
5	GWNI	13.9
6	YVEEL	0.799
7	MHIRL	0.306
8	SALAM	2.66
9	WYSLA	4.59
10	AGWNI	8.55
11	LGFEY	9.79
12	GWNIP	6.19
13	WYSLAM	7
14	AGWNIP	7.64
15	LGFEYY	7.25
16	VIPMGL	2.89
17	AGWNIPIGT	5.25
18	LSKAQSDFG	-1.4
19	YAEERYPIL	3.8
20	LVEKGDVAFI	-0.07
21	WYSLAMAASDI	2.621
22	IEWEGIESGSVEQA	0.73
23	AIEWEGIESGSVEQA	0.95
24	IAAEVYEHTEGSTTSY	2.39
25	PIAAEVYEHTEGSTTSY	1.68
26	IANNEADAISLDGGQVFEAG	0.43

## Supplementary Materials

E-supplementary data of this work can be found in https://www.mdpi.com/1660-3397/17/11/633/s1. Table S1. Primer sequences of target and reference genes. Table S2. EHP identified by HPLC-MS/MS and the antioxidant activity of peptides in the QSAR model. Table S3. Distribution of peptides with different molecular weights.
