# Supplementary material for: Quantitative Structure-Activity Relationship Model to Predict Antioxidant Effects of the Peptide Fraction Extracted from a Co-Culture System of Chlorella pyrenoidosa and Yarrowia lipolytica
Source: Mar Drugs. 2019 Nov 8;17(11):633. doi: 10.3390/md17110633 (PMC6891513; doi:10.3390/md17110633)
Supplement: Supplementary file 1 [file marinedrugs-17-00633-s001.zip › marinedrugs-624288 suppl/Table S1.pdf]

Table S1. Primer sequences of target and reference genes

The name of primer	sequence
SOD-F	TCTATCCAGAAAACACG
SOD-R	ATTACACCACAAGCCAA
CAT-F	ATGGGGAGGCAGTTTATT
CAT-R	GAGAGGGTAGTCCTTGTG
GSHPx-F	GAGAACGCCAAGAACGAA
GSHPx-R	CGGAGACCAGGTGATGAG
Nrf2-F	GCGACGGAAAGAGTATGA
Nrf2-R	ACTGGATGTGCTGGGCTG
KEAP1-F	GGGCGAGAAGTGTGTC
KEAP1-R	CAGCGAAGTTGGCGAT
GAPDH-F	CCACTCCTCCACCTTTG
GAPDH-R	CACCACCCTGTTGCTGT
