# Supplementary material for: Quantitative Structure-Activity Relationship Model to Predict Antioxidant Effects of the Peptide Fraction Extracted from a Co-Culture System of Chlorella pyrenoidosa and Yarrowia lipolytica
Source: Mar Drugs. 2019 Nov 8;17(11):633. doi: 10.3390/md17110633 (PMC6891513; doi:10.3390/md17110633)
Supplement: Supplementary file 1 [file marinedrugs-17-00633-s001.zip › marinedrugs-624288 suppl/Table S2.pdf]

**Table S2.** EHP identified by HPLC/MSMS EHP identified using HPLC/MSMS and the antioxidant activity of peptides in the QSAR model

| Peptides Sequence | Molecular Mass (Da) | Charged ion(m/z) | Retention Time(min) | Activity |
|---|---|---|---|---|
| YEDN(+.98)GDAVN(+.98)NLVVLVQK | 1890.921 | 946.4719 | 26.42 | 3.094416 |
| ADLEM(+15.99)QIESLTEELAYLKK | 2239.129 | 747.3864 | 45.78 | 2.357068 |
| VLYDAEISQIHQSVTDTNVILSMDNSR | 3047.487 | 1016.839 | 34.56 | 4.41314 |
| TSQNSELNNMQDLVEDYKK | 2255.038 | 752.6888 | 23.99 | 2.5751 |
| DIENQYETQITQIEHEVSSSGQEVQSSAK | 3263.507 | 1088.844 | 36.16 | 2.21429 |
| LLAELEGKDIN(+.98)EVIAAGTAK | 2055.11 | 686.0473 | 31.03 | 5.288311 |
| YEDN(+.98)GDAVNNLVVLVQK | 1889.937 | 945.9794 | 29.42 | 3.094416 |
| VPTPTVSVVDLVVQVEK | 1808.03 | 905.0219 | 34.2 | 2.1276 |
| AGAEIFGDDGIQYLGIPGLINAK | 2331.211 | 778.0784 | 43.97 | 5.450156 |
| YEDN(+.98)GDAVNN(+.98)LVVLVQK | 1890.921 | 946.4711 | 33.42 | 3.094416 |
| VPTLDVSVVDLTVNLDKPAK | 2122.189 | 708.3974 | 34.4 | 3.321271 |
| THNLEPYFESFINNLR | 1992.969 | 665.3312 | 39.17 | 4.855789 |
| AAGIEVEPYWPALFAK | 1760.914 | 587.9805 | 40.74 | 4.179178 |
| TITLEVESSDTIENVK | 1776.899 | 889.4581 | 22.27 | 4.113249 |
| VVAAYLLAALGGN(+.98)AAPAEADIKK | 2226.226 | 743.0765 | 35.19 | 1.201185 |
| LPAGVWEDVLAAVK | 1466.813 | 734.4136 | 42.82 | 1.872748 |
| LLAELEGKDINEVIAAGTAK | 2054.126 | 685.7175 | 28.91 | 5.288311 |
| ISTVQEAADLIAAQMDK | 1802.908 | 601.9787 | 41.49 | 3.345185 |
| WAM(+15.99)LGALGC(+57.02)VTPELLQR | 1929.981 | 644.3336 | 36.32 | 5.166186 |
| ISTVQ(+.98)EAADLIAAQM(+15.99)DK | 1819.887 | 910.9534 | 31.72 | 3.345185 |
| NVSTGDVNVEMNAAPGVDLTQLLNNMR | 2871.386 | 958.1385 | 44.16 | 4.228911 |
| GPFDPLGLADDPEVLAELK | 1995.02 | 666.0156 | 44.92 | 3.788655 |
| HGVQELEIELQSQLSK | 1836.958 | 919.4897 | 28.47 | 5.655108 |
| SDLEMQYETLQEELMALKK | 2298.112 | 767.0458 | 37.57 | 1.576727 |
| TTFDVIIEDVPADKR | 1717.889 | 573.6396 | 24.15 | 2.044679 |
| RM(+15.99)PTFFETFPVVLLDKDGVVR | 2481.309 | 621.3347 | 37.78 | 6.526337 |
| FDLNAVADTLPK | 1302.682 | 652.3487 | 27.39 | 1.461321 |
| GGGGGGYGSGGSSYGSGGGSYGSGGGGG GGR | 2382.945 | 795.3353 | 5.7 | 8.268596 |
| VAAASVLDVQEATDK | 1515.778 | 758.8984 | 14.67 | 1.713609 |
| EIETYHNLLEGGQEDFESSGAGK | 2509.125 | 837.3909 | 23.04 | 4.622056 |
| NVQALEIELQSQLALK | 1796.004 | 599.6773 | 37.45 | 2.825362 |
| FLEQQNQVLQTK | 1474.778 | 738.4012 | 12.64 | 2.994335 |
| DTDSEAELLEAFK | 1466.678 | 734.3491 | 33.24 | 2.349638 |
| TAAELLQDKPLLK | 1438.84 | 720.4319 | 16.87 | 3.166215 |
| M(+15.99)PTFFETFPVVLLDKDGVVR | 2325.208 | 776.0796 | 42.65 | 6.096686 |

| | | | | |
|---|---|---|---|---|
| VDLLNQEIEFLK | 1459.792 | 730.9026 | 36.79 | 4.084672 |
| FDLN(+.98)AVADTLPK | 1303.666 | 652.8412 | 27.89 | 1.461321 |
| AVNVGTVFQVGDGIAR | 1601.853 | 534.9602 | 28.41 | 5.692646 |
| LLAGLDDLALDIPDAAR | 1750.946 | 876.4788 | 38.75 | 3.776844 |
| IVAVTGEEAQAAIALADQLAAQVEAAGK | 2707.439 | 903.4813 | 50.22 | 2.414049 |
| N(+.98)GAANFGEAVWFK | 1410.657 | 706.3381 | 34.94 | 5.845443 |
| YSEM(+15.99)VFPILAPDPAK | 1692.843 | 847.4319 | 29.45 | 3.628622 |
| ELTTEIDNNIEQISSYK | 1995.964 | 998.99 | 30.52 | 2.660309 |
| YC(+57.02)VQLSQIQAQISALEEQLQQIR | 2745.412 | 916.1466 | 49.47 | 4.116221 |
| FDIQLDEEGAEK | 1392.641 | 697.3323 | 18.89 | 3.381736 |
| N(+.98)GVANFGEAVWFK | 1438.688 | 720.3521 | 38.49 | 5.910272 |
| SLNNQFASFIDK | 1382.683 | 692.3502 | 27.11 | 2.425302 |
| WLGPFSEGSVPSYLK | 1665.84 | 833.9282 | 34.98 | 4.232334 |
| VAAASVLDVQEATDKK | 1643.873 | 548.9678 | 10.95 | 0.699534 |
| FVGYDPDAKEFDAEILR | 1983.958 | 662.335 | 27.04 | 3.937038 |
| GEFPGDYGWDTAGLSADPETFAR | 2458.071 | 820.3682 | 34.98 | 5.650221 |
| IINEPTAAAIAYGLDK | 1658.888 | 553.9728 | 25.98 | 4.298241 |
| TLEVAEATWAETFK | 1594.788 | 798.4015 | 28.46 | 3.087761 |
| LNDLEDALQQAK | 1356.689 | 679.3544 | 20.72 | 5.553867 |
| LLEGEDAHLSSSQFSSGSQSSR | 2308.057 | 770.3701 | 12.29 | 3.339777 |
| AGASIFN(+.98)EDGLN(+.98)YLGN | 1655.731 | 828.8729 | 37.61 | 6.03802 |
| VAGVDDVTGEPLVK | 1397.74 | 699.8749 | 15.82 | 2.97495 |
| NQILNLTTDNANILLQIDNAR | 2366.255 | 789.7602 | 39.43 | 4.937128 |
| ADLEMQIESLTEELAYLKK | 2223.134 | 742.0527 | 45.36 | 2.357068 |
| VLM(+15.99)LADGSAVFAK | 1336.706 | 669.3613 | 23.21 | 4.351017 |
| TTFDVIIEDVPADK | 1561.788 | 781.9026 | 28.95 | 1.443551 |
| IKFEMEQNLR | 1306.67 | 436.5681 | 14.12 | 5.559506 |
| RIDADLDAYIAK | 1362.714 | 455.2481 | 18.25 | 4.500611 |
| MIVELIQPIAIEK | 1495.868 | 748.9407 | 33.98 | 2.549794 |
| M(+15.99)IVELIQPIAIEK | 1511.863 | 756.9407 | 29.73 | 2.549794 |
| GFFDPLGLADDPDTLAELK | 2032.999 | 1017.505 | 45.87 | 4.272106 |
| IRPAVQEDGGDIVFR | 1670.874 | 557.9699 | 15.18 | 2.636075 |
| VAAASVLDVQ(+.98)EATDKK | 1644.857 | 549.297 | 13.67 | 0.699534 |
| YEDN(+.98)GDAVNN(+.98)LVVLVQ(+.98)K | 1891.905 | 946.9615 | 36.55 | 3.094416 |
| ISTVQEAADLIAAQM(+15.99)DK | 1818.903 | 607.3094 | 30.81 | 3.345185 |
| TIDDLKNQILNLTTDNANILLQIDNAR | 3051.62 | 1018.212 | 47.55 | 4.531282 |
| VAAASVLDVQ(+.98)EATDK | 1516.762 | 759.3928 | 16.17 | 1.713609 |
| GSLGGGFSSGGFSGGSFSR | 1706.765 | 854.3991 | 21.43 | 3.826548 |
| QAGFDIIYEAR | 1281.635 | 641.8286 | 24.51 | 4.602492 |
| TVLIM(+15.99)ELINNVAK | 1472.827 | 737.4205 | 37.97 | 3.276097 |
| GGSGGSHGGGSGFGGESGGSYGGGEEASG SGGGYGGGSGK | 3222.274 | 1075.105 | 6.37 | 8.254469 |
| IAQIPVGDAFLGR | 1355.756 | 452.9261 | 27.63 | 5.432212 |
| VVAWYDN(+.98)EWGYSQR | 1772.779 | 887.3998 | 28.26 | 3.05539 |

| | | | | |
|---|---|---|---|---|
| GEYPGDYGWDTAGLSADPETFAR | 2474.066 | 825.6995 | 32.49 | 5.466883 |
| SM(+15.99)AILNSFIVDQFEK | 1756.871 | 879.4434 | 36.19 | 4.101922 |
| IDADLDAYIAK | 1206.613 | 604.3152 | 22.15 | 3.223599 |
| AGAQIFQEGGLDYLGN | 1651.784 | 826.9021 | 33.7 | 6.067209 |
| ALEESNYELEGK | 1380.641 | 691.332 | 10.96 | 3.540331 |
| VVAAYLLAALGGN(+.98)AAPAEADIK | 2098.131 | 700.3829 | 41.78 | 1.687362 |
| N(+.98)GVAN(+.98)FGEAVWFK | 1439.672 | 720.8442 | 40.42 | 5.910272 |
| IIN(+.98)EPTAAAIAYGLDKK | 1787.967 | 596.9933 | 23.87 | 1.235513 |
| RM(+15.99)PTFFETFPVVLLDK | 1955.023 | 652.6808 | 40.17 | 3.456669 |
| WAM(+15.99)LGALGC(+57.02)IFPEVLEK | 1948.979 | 975.4975 | 45.51 | 4.126597 |
| VVDALGNPIDGK | 1196.64 | 599.3318 | 14 | 2.026823 |
| WAM(+15.99)LGALGC(+57.02)VTPELLQ(+.98)R | 1930.965 | 966.4913 | 37.33 | 5.166186 |
| Q(+.98)AGFDIIYEAR | 1282.619 | 642.3194 | 25.99 | 4.602492 |
| LGVQEGFVNVLSR | 1416.773 | 709.3933 | 28.28 | 5.579679 |
| HGVQELEIELQSQLSKK | 1965.053 | 656.0252 | 25.14 | 4.119127 |
| KPDFDAYIDPQKK | 1563.793 | 522.2762 | 12.03 | 2.676661 |
| DIELVM(+15.99)TQAGVSR | 1433.718 | 717.8683 | 15.12 | 5.074851 |
| PSKEQ(+.98)DFPGVVLR | 1471.767 | 491.5994 | 16.22 | 2.225238 |
| FADPADPLLFSVR | 1446.751 | 724.3848 | 32.27 | 4.63996 |
| GIRPAINVGLSVSR | 1437.842 | 480.2879 | 13.25 | 3.045506 |
| GIRPAINVGISVSR | 1437.842 | 480.2879 | 13.25 | 3.045506 |
| NLDLDSIIAEVK | 1328.719 | 665.3673 | 38.42 | 4.744527 |
| N(+.98)GAAN(+.98)FGEAVWFK | 1411.641 | 706.8302 | 37.17 | 5.845443 |
| AATVGDIIIAVVK | 1268.77 | 635.393 | 38.1 | 3.968148 |
| SPTGEIIFGGETM(+15.99)R | 1509.713 | 755.869 | 17.81 | 2.818424 |
| GDSLYPGGFFDPLGLADDPDTLAELK | 2722.301 | 908.4451 | 47.72 | 3.866052 |
| IAFLDGN(+.98)TAVM(+15.99)LGK | 1465.749 | 733.8842 | 27.97 | 5.298836 |
| ADLEMQIESLTEELAYLK | 2095.04 | 699.3534 | 49.92 | 3.649302 |
| EFQREDFEGIFTAM(+15.99)DGLPVTIR | 2586.242 | 863.0917 | 35.94 | 2.515433 |
| NIFVVGPVPGK | 1125.655 | 563.8364 | 22.08 | 3.246092 |
| VTGEDSFSIN(+.98)GK | 1253.578 | 627.8016 | 12.6 | 1.521659 |
| SLDLDSIIAEVK | 1301.708 | 651.8607 | 38.57 | 4.272328 |
| EIKFEFETIDTL | 1483.745 | 742.8812 | 35.79 | 0.976619 |
| FNLGTESLSAGVTWK | 1608.815 | 805.412 | 24.97 | 1.427317 |
| SLQEALASELAAR | 1357.72 | 453.584 | 28.34 | 4.27031 |
| ILSSVGIEAEAER | 1372.72 | 687.3702 | 15.37 | 4.140952 |
| LAGDYGFDPLGLGVDSDR | 1865.88 | 933.9521 | 35.3 | 3.042197 |
| VEM(+15.99)ALYAFGFN(+.98)GDN | 1563.655 | 782.8361 | 36.33 | 2.619119 |
| IRPDEISSIIK | 1269.729 | 424.2534 | 14.44 | 3.886637 |
| TISDDLEFLISQAVDPK | 1889.962 | 630.9968 | 50.38 | 1.183225 |
| RAQLGEIFEFDR | 1479.747 | 494.2596 | 25.66 | 5.677311 |
| LNDLEEALQQAK | 1370.704 | 686.3612 | 22.92 | 5.553867 |
| YEDNGDAVNNLVVLVQK | 1888.953 | 945.475 | 28.36 | 3.094416 |

| | | | | |
|---|---|---|---|---|
| IDADLDAYIAKAYWTEGR | 2070.006 | 691.0098 | 31.2 | 2.643528 |
| AVNVGTVFQ(+.98)VGDGIAR | 1602.837 | 802.4216 | 26.31 | 5.692646 |
| ARQAGFDIIYEAR | 1508.774 | 503.9352 | 17.97 | 4.42238 |
| LNDLEDALQQAKEDLAR | 1940.98 | 648.003 | 34.54 | 6.533206 |
| DVPADKFIEAFAAYLK | 1796.935 | 599.9875 | 48.63 | 3.762379 |
| PSKEQDFPGVVLR | 1470.783 | 491.2716 | 15 | 2.225238 |
| LAADVAGTPTVLIAR | 1466.846 | 734.4297 | 21.92 | 4.466387 |
| YFEVILVDPAHK | 1429.761 | 477.5967 | 25.23 | 1.788579 |
| GILAVSDEPLVSC(+57.02)DYR | 1792.867 | 897.4388 | 27.02 | 2.380411 |
| IVEVLLEKESM(+15.99)TGDEFR | 2009.998 | 671.0121 | 20.89 | 2.775159 |
| NYSPYYNTIDDLKDQIVDLTVGNNK | 2901.403 | 968.1439 | 43.59 | 5.152757 |
| AAALNIVPTTTGAAK | 1397.788 | 699.9051 | 12.92 | 5.277548 |
| TVLIM(+15.99)ELINNIAK | 1486.843 | 496.6234 | 42.45 | 3.547136 |
| LALDLEIATYR | 1276.703 | 639.3532 | 30.64 | 3.106286 |
| LALDIEIATYR | 1276.703 | 639.3532 | 30.64 | 3.106286 |
| VVLVDDLIATGGTLR | 1540.882 | 771.4471 | 34.58 | 4.929078 |
| SFVEVGLLGGDASR | 1405.72 | 703.8669 | 26.91 | 3.053346 |
| DFTAQIQTLDIPGEVK | 1773.915 | 887.9647 | 31.22 | 4.87734 |
| LGAN(+.98)VASAQGPTGLGK | 1440.757 | 721.3848 | 11.62 | 7.971924 |
| ADDPLAAKLN(+.98)DVEDVER | 1869.896 | 624.3134 | 21.34 | 3.480283 |
| ELEDLDAVLAELGLEAK | 1826.951 | 914.4818 | 49.15 | 3.598534 |
| IGDTVELVGLR | 1170.661 | 586.3394 | 22.43 | 6.523536 |
| FTQAGSEVSALLGR | 1434.747 | 718.3785 | 23.14 | 3.942627 |
| WN(+.98)PSKEQ(+.98)DFPGVVLR | 1772.873 | 591.968 | 26.51 | 4.253433 |
| KGDLNVGAVLILPEGFELAPPDRIPEEM(+15.99)K | 3162.663 | 791.6749 | 36.39 | 7.172476 |
| KGDLNVGAVLILPEGFEIAPPDRIPEEM(+15.99)K | 3162.663 | 791.6749 | 36.39 | 7.172476 |
| FLFVAEAIYK | 1199.659 | 600.8375 | 32.98 | 3.613396 |
| QFSAEEISSM(+15.99)VLSK | 1570.755 | 786.3859 | 25.61 | 2.535905 |
| ALSLSEISVLLEQK | 1528.871 | 765.4446 | 40.61 | 3.191908 |
| FVQAGSEVSALLGR | 1432.767 | 717.3934 | 25.46 | 4.094015 |
| KGDLNVGAVLILPEGFELAPPDR | 2419.311 | 807.4431 | 39.32 | 6.671945 |
| KGDLNVGAVLILPEGFEIAPPDR | 2419.311 | 807.4431 | 39.32 | 6.671945 |
| LLFEVAPLALLVEK | 1553.943 | 777.9788 | 48.35 | 3.970777 |
| ILRPESYWFR | 1365.719 | 456.248 | 21.54 | 5.01801 |
| IVTGVPDAIPGVGPALVELLR | 2085.22 | 696.0765 | 47.78 | 4.170556 |
| TVLIM(+15.99)ELIN(+.98)NVAK | 1473.811 | 737.9101 | 28.53 | 3.276097 |
| LYFQPYSAEK | 1244.608 | 623.3143 | 17.21 | 3.489219 |
| EQIFEM(+15.99)PTGGAAIM(+15.99)R | 1681.78 | 841.9044 | 14.99 | 4.015168 |
| NKLNDLEDALQQAK | 1598.826 | 533.9537 | 22.63 | 3.920521 |
| DINEVIAAGTAK | 1200.635 | 601.3284 | 17.17 | 4.669747 |
| VVLAYSGGLDTSVILK | 1633.929 | 817.9709 | 30.3 | 1.603292 |
| SLYPGGFFDPLGLADDPDTLAELK | 2550.253 | 1276.135 | 48.08 | 3.309771 |

| | | | | |
|---|---|---|---|---|
| QISNLQQSISDAEQR | 1715.844 | 858.9324 | 17.01 | 2.558379 |
| NVSTGDVNVEM(+15.99)NAAPGVDLTQLLNNMR | 2887.38 | 963.472 | 41.19 | 4.228911 |
| AVN(+.98)VGTVFQVGDGIAR | 1602.837 | 535.2877 | 29.4 | 5.692646 |
| LGANVASAQGPTGLGK | 1439.773 | 720.8939 | 10.61 | 7.971924 |
| FYPEEEKN(+.98)DAEFAK | 1716.752 | 573.2619 | 11.51 | 5.462216 |
| VLDITDDDLGNLITAGLR | 1913.011 | 957.5108 | 41.86 | 5.160092 |
| EADQDGDGQVDYSEFVK | 1900.796 | 951.4108 | 19.53 | 5.524653 |
| WAM(+15.99)LAAAGAIIPEGLAAN(+.98)GADVK | 2226.136 | 743.0586 | 34.44 | 4.456899 |
| AGAGSATLSM(+15.99)AYAAAR | 1483.709 | 742.8612 | 11.7 | 5.750762 |
| QAGFDIIYEARDLDLPQGVR | 2275.16 | 759.4017 | 32.27 | 4.623152 |
| TVLIMELINNVAK | 1456.832 | 729.4238 | 45.5 | 3.276097 |
| YDDPVLLAADVEK | 1446.724 | 724.3715 | 25.92 | 3.095543 |
| TGTIVDVPVGK | 1084.613 | 543.3126 | 13.32 | 6.088117 |
| SIGIN(+.98)EFGASAPGPLLYEK | 1962.994 | 982.4994 | 32.39 | 3.128158 |
| ARQ(+.98)AGFDIIYEAR | 1509.758 | 504.2629 | 19.72 | 4.42238 |
| AVN(+.98)VTGPN(+.98)GAPPEGAPR | 1604.779 | 803.4003 | 8.93 | 4.530311 |
| LALDVEIATYR | 1262.687 | 632.3467 | 26.86 | 3.106286 |
| FLFVAEAIYKSQAETGEIK | 2143.12 | 715.374 | 31.14 | 6.179964 |
| LLFELGGIPER | 1242.697 | 622.3583 | 32.41 | 4.027011 |
| LKYENEVALR | 1233.672 | 412.2334 | 9.78 | 4.315253 |
| ATAELKN(+.98)GEVLLLENVR | 1869.021 | 624.0143 | 24.54 | 3.916233 |
| IAFLDGNTAVMLGK | 1448.77 | 725.3978 | 31.03 | 5.298836 |
| DIELVM(+15.99)TQ(+.98)AGVSR | 1434.702 | 718.3617 | 16.41 | 5.074851 |
| GDLNVGAVLILPEGFELAPPDRIPEEM(+15.99)K | 3034.568 | 1012.528 | 40.48 | 2.443667 |
| GDLNVGAVLILPEGFEIAPPDRIPEEM(+15.99)K | 3034.568 | 1012.528 | 40.48 | 2.443667 |
| LAVFM(+15.99)EWVDSFR | 1514.723 | 758.369 | 43.54 | 3.975643 |
| VVDALGNPIDGKGPLK | 1591.893 | 531.6401 | 13.91 | 4.58763 |
| VVDALGNPIDGKGPIK | 1591.893 | 531.6401 | 13.91 | 4.684391 |
| YEELQVTVGR | 1192.609 | 597.3148 | 13.86 | 4.592395 |
| GADVVIIPAGVPR | 1262.735 | 632.3731 | 22.12 | 6.737946 |
| ADLEM(+15.99)QIESLTEELAYLK | 2111.034 | 704.6871 | 50.14 | 3.649302 |
| VVAWYDN(+.98)EWGYSQ(+.98)R | 1773.763 | 887.8992 | 29.13 | 3.05539 |
| YDSTM(+15.99)GTFAADVK | 1420.618 | 711.3145 | 11.73 | 2.874879 |
| LPTIAAAIYR | 1087.639 | 544.8277 | 23.76 | 3.428936 |
| YNLSLGIGLADVK | 1361.755 | 681.8834 | 30.11 | 3.284175 |
| EC(+57.02)LPLILLLR | 1238.742 | 620.3779 | 44.32 | 3.752701 |
| IAFLDGNTAVM(+15.99)LGK | 1464.765 | 733.3837 | 27.3 | 5.298836 |
| VAERTISDDLEFLISQAVDPK | 2345.211 | 782.7517 | 42.65 | 0.348945 |
| EAYPGDVFYLHSR | 1552.731 | 518.5873 | 20.11 | 4.228579 |
| GC(+57.02)IVEVDAAPFK | 1304.643 | 653.3377 | 21.17 | 2.401757 |

| | | | | |
|---|---|---|---|---|
| VGKN(+.98)N(+.98)IDELGSPDK | 1486.715 | 496.5824 | 9.85 | 3.773616 |
| ELDYLDGAVSAPK | 1376.682 | 689.3525 | 19.8 | 3.679375 |
| EISPEILNAIR | 1253.698 | 627.8564 | 27.92 | 4.499454 |
| ISTVQ(+.98)EAADLIAAQMDK | 1803.892 | 602.3048 | 42.6 | 3.345185 |
| VGWKPDFVEK | 1203.629 | 402.2195 | 14.79 | 4.810115 |
| ETAEVEYLTAR | 1280.625 | 641.3226 | 14.62 | 3.233485 |
| KNIFVVGPVPGK | 1253.75 | 418.9268 | 15.21 | 3.669901 |
| AGEGFALLIPSK | 1201.671 | 601.8414 | 29.19 | 4.028118 |
| FDLNAVADTLPKAAK | 1572.851 | 525.2927 | 24.67 | 2.328703 |
| WNPSKEQDFPGVVLR | 1770.905 | 591.3167 | 25.34 | 4.253433 |
| SQYEQLAEQNR | 1364.632 | 683.3286 | 7.97 | 4.39434 |
| IQAGEM(+15.99)VEFGSGVR | 1494.714 | 748.3702 | 13.48 | 3.227871 |
| KYSEM(+15.99)VFPILAPDPAK | 1820.938 | 607.9864 | 22.89 | 4.625483 |
| VAAASVLDVQ(+.98)EATDKKGK | 1829.973 | 458.5043 | 10.17 | 3.912469 |
| VGLTALTM(+15.99)AEYFR | 1486.749 | 744.3817 | 27.01 | 6.989433 |
| DIELVMTQAGVSR | 1417.724 | 709.8718 | 24.23 | 5.074851 |
| M(+15.99)VADFVQFLYR | 1403.691 | 702.8544 | 37.35 | 2.899641 |
| FEAQVYVLTK | 1196.644 | 599.3278 | 19.73 | 2.108022 |
| TNAENEFVTIK | 1264.63 | 633.3265 | 14.52 | 2.718438 |
| VGKN(+.98)NIDELGSPDK | 1485.731 | 496.2541 | 8.63 | 3.773616 |
| YEDN(+.98)GDAVN(+.98)NLVVLVQ(+.98)K | 1891.905 | 631.6459 | 28.91 | 3.094416 |
| EGIRPDVFLLINVPDELLIDR | 2435.342 | 812.7874 | 47.96 | 4.987835 |
| ASSIAQVVNTLQER | 1514.805 | 758.4043 | 26.59 | 4.297266 |
| GVEFVLPTDVIVADK | 1600.871 | 801.4386 | 36.4 | 1.963353 |
| TAIAIDAIINQK | 1269.729 | 635.8713 | 24.93 | 1.493575 |
| LIESPAPGIISR | 1251.719 | 626.8691 | 16.34 | 2.419496 |
| DAGAIAGLEVLR | 1183.656 | 592.8378 | 28.06 | 3.905588 |
| IYLDISDEIK | 1207.634 | 604.826 | 24.82 | 3.598243 |
| SGGGFSSGSAGIINYQR | 1656.786 | 829.3972 | 15.75 | 6.363785 |
| VLDELTLTK | 1030.591 | 516.3032 | 17.55 | 3.540145 |
| LDGLDAVLSK | 1029.571 | 515.7932 | 22.32 | 1.768684 |
| IDM(+15.99)VEDEELVELVEM(+15.99)ELR | 2222.033 | 741.684 | 47.53 | 3.971875 |
| LYDALSPFIR | 1193.644 | 597.8312 | 31.73 | 5.580448 |
| ELQDIIAILGLDELSEEDRLIVAR | 2722.475 | 908.5002 | 49.65 | 4.127458 |
| IIN(+.98)EPTAAAIAYGLDK | 1659.872 | 554.3021 | 28.97 | 4.298241 |
| GM(+15.99)M(+15.99)PGILEM(+15.99)LER | 1423.651 | 712.8366 | 23.42 | 5.203787 |
| S(+42.01)KSELAC(+57.02)VYAVLALADDGL EVTPENIVTLTK | 3360.737 | 1121.268 | 50.48 | 3.421232 |
| ITIGLYGDEVPK | 1303.702 | 652.8549 | 22.82 | 1.37053 |
| VSGVSLLALFR | 1160.692 | 581.3511 | 41.42 | 2.745646 |
| DLGGYDKFVEAFK | 1487.73 | 496.9191 | 31.3 | 1.920327 |
| TLDESVAGDNVGILLR | 1670.884 | 836.4393 | 28.4 | 4.769502 |
| EAQEEVAYEIAEAVISALK | 2062.047 | 688.359 | 53.64 | 3.777793 |
| YEDNGDAVN(+.98)N(+.98)LVVLVQ(+.98)K | 1891.905 | 946.9598 | 40.15 | 3.094416 |

| | | | | |
|---|---|---|---|---|
| VLGVTTLDVVR | 1170.697 | 586.3565 | 23.94 | 3.985761 |
| AFDEEALADLVALK | 1503.782 | 752.8989 | 40.43 | 3.007116 |
| VLYDAEISQIHQSVTDTNVILSM(+15.99)DNSR | 3063.482 | 1022.153 | 31.22 | 4.41314 |
| KEQC(+57.02)LALLTQLR | 1471.818 | 491.6158 | 21.83 | 4.474069 |
| VVAGPGDKPM(+15.99)IEVEFK | 1730.891 | 577.9755 | 15.43 | -0.05956 |
| EFAEFVSQLR | 1224.614 | 613.3164 | 27.67 | 4.211858 |
| GFAAAAVEEIVK | 1203.65 | 602.8303 | 27.43 | 4.152566 |
| FELGYYGLKPDIK | 1541.813 | 514.9462 | 25.17 | 2.001234 |
| YEVLTRSADGDEG | 1410.626 | 706.3284 | 10.19 | 2.318827 |
| AFAEYKN(+.98)GSLM(+15.99)EDDAK | 1804.783 | 602.6055 | 9.8 | 2.821639 |
| QSLEASLAETEGR | 1389.674 | 464.2374 | 15.66 | 3.231574 |
| TISDDLEFLISQ(+.98)AVDPK | 1890.946 | 946.483 | 49.47 | 1.183225 |
| AIGAELDLTDKGLGVR | 1626.894 | 543.3089 | 22.74 | 2.95241 |
| VVDLLAPYR | 1044.597 | 523.3089 | 25.73 | 3.037776 |
| VEIESLVEGVDLSEPLTR | 1984.036 | 662.3507 | 38.14 | 3.251213 |
| AVN(+.98)VGTVFQ(+.98)VGDGIAR | 1603.821 | 535.618 | 30.63 | 5.692646 |
| AGAEIFGDDGIQYLGIPGLIN(+.98)AK | 2332.195 | 1167.104 | 44.73 | 5.450156 |
| IVDWLAEDFQK | 1362.682 | 682.3457 | 30.85 | 4.073024 |
| EIEIDIEPTDTVQR | 1656.821 | 829.4143 | 22.06 | 2.82458 |
| LGLDVTVIEGR | 1170.661 | 586.3333 | 25.45 | 6.114502 |
| PFSEGSVPSYLK | 1309.655 | 655.8383 | 19.7 | 4.286531 |
| LEEQ(+.98)KGVDILLAAIK | 1639.94 | 547.6566 | 31.98 | 3.188458 |
| LEEQ(+.98)KGVDILLAALK | 1639.94 | 547.6566 | 31.98 | 3.091697 |
| LSDEILPLLSELR | 1496.845 | 499.953 | 43.92 | 4.413822 |
| AQLGEIFEFDR | 1323.646 | 662.8334 | 31.67 | 3.713689 |
| VN(+.98)YAGVSTN(+.98)N(+.98)YALDEVEEVKA | 2287.038 | 1144.526 | 28.85 | 1.759802 |
| ADLNVPLDGDLK | 1268.661 | 635.3391 | 20.92 | 3.52292 |
| IGM(+15.99)PDVEEVISAELDYK | 1922.918 | 641.9815 | 38.03 | 4.605637 |
| SYWVDLLADSVEK | 1523.751 | 762.8864 | 40.32 | 3.529162 |
| AVVTVPAYFN(+.98)DSQR | 1566.768 | 784.3918 | 23.21 | 2.249741 |
| EIIIDVPLAWK | 1295.749 | 648.8829 | 37.5 | 2.176313 |
| GM(+15.99)QEFAGALLAR | 1278.639 | 640.3314 | 20.46 | 5.649725 |
| LEQEQQFLIAK | 1345.724 | 673.8693 | 17.28 | 4.089113 |
| DTFYLYSAIVAAGLER | 1787.909 | 596.9787 | 46.46 | 6.006249 |
| EVTLGFVDLM(+15.99)R | 1294.659 | 648.3389 | 27.75 | 4.531163 |
| FM(+15.99)QIFDEIYR | 1376.643 | 689.332 | 28.35 | 4.551404 |
| Q(-17.03)SLEASLAETEGR | 1372.647 | 687.3308 | 24.4 | 3.231574 |
| LLFELGGIPERELEK | 1741.961 | 581.6648 | 30.25 | 5.020274 |
| YEELQITAGR | 1178.593 | 590.3078 | 13.3 | 4.465108 |
| FDPLGLADDPDTLAELK | 1828.909 | 915.4619 | 38.37 | 3.144524 |
| SDLSILFK | 921.5171 | 461.7692 | 28.44 | 1.106054 |
| GGAFTGELSAEM(+15.99)LK | 1425.681 | 713.8526 | 18.68 | 8.075564 |

| | | | | |
|---|---|---|---|---|
| DLDLPQGVR | 1011.535 | 506.7784 | 15.98 | 5.709387 |
| KARQ(+.98)AGFDIIYEAR | 1637.853 | 410.474 | 14.72 | 4.571433 |
| WELLQQVDTSTR | 1474.742 | 738.3823 | 26.06 | 3.665902 |
| NKLNDLEEALQQAK | 1612.842 | 538.623 | 24.36 | 3.920521 |
| GGEM(+15.99)VPFM(+15.99)FTVK | 1373.636 | 687.8281 | 24.15 | 6.943757 |
| GTFDRPALLAR | 1215.672 | 406.235 | 13.91 | 5.190312 |
| DINVVLVAPK | 1066.639 | 534.3257 | 21.13 | 1.38441 |
| LGEIPGAIPK | 993.5858 | 497.8023 | 14.65 | 5.982169 |
| ISTVQEAADLIAAQ(+.98)M(+15.99)DK | 1819.887 | 607.6385 | 33.23 | 3.345185 |
| KARQAGFDIIYEAR | 1636.868 | 546.6349 | 13.57 | 4.571433 |
| VN(+.98)GGPAGEGLDALYPGEAFDPLGLA DDPDTFAELK | 3531.657 | 1178.229 | 47.83 | 2.195128 |
| ISAALAEVVQR | 1155.661 | 578.8375 | 16.72 | 1.53425 |
| VAVELSPYDLR | 1260.671 | 631.3371 | 22.71 | 4.161007 |
| EQIATFQQELGK | 1390.709 | 696.3587 | 17.36 | 4.129311 |
| VIGGGLPVGAYGGK | 1243.692 | 622.8549 | 16.06 | 2.22931 |
| ILSEEFGWDKELAK | 1663.846 | 555.624 | 24.15 | 4.956772 |
| Q(-17.03)AGFDIIYEAR | 1264.609 | 633.3134 | 35.22 | 4.602492 |
| EFLNLPSEIVPATLK | 1669.929 | 835.9726 | 37.36 | 2.592886 |
| TAAELLQ(+.98)DKPLLK | 1439.824 | 720.9222 | 18.41 | 3.166215 |
| KADM(+15.99)IIQVLPTQLVPDEK | 2053.113 | 685.3787 | 27.44 | 3.411015 |
| SDTYVIFGEAK | 1228.598 | 615.307 | 20.36 | 4.682886 |
| VVDLLAPYQK | 1144.649 | 573.3335 | 22.64 | 3.238636 |
| DRFLFVAEAIYK | 1470.787 | 491.2708 | 34.75 | 3.513485 |
| M(+15.99)PTFFETFPVVLLDK | 1798.922 | 900.4662 | 46.33 | 3.027018 |
| FYPEEEKN(+.98)DAEFAKK | 1844.847 | 462.2237 | 8.83 | 2.68075 |
| EGAVLC(+57.02)FDEVM(+15.99)TGFR | 1745.775 | 873.9014 | 29.77 | 5.71701 |
| QIIEGGEFFVLK | 1378.75 | 690.3847 | 32.69 | 2.940485 |
| GEFPGDYGWDTAGLSADPETFARYR | 2777.236 | 926.7536 | 31.9 | 4.894365 |
| AGYSPIGFVR | 1065.561 | 533.7886 | 20.14 | 9.726983 |
| VVVVDEFLGR | 1131.629 | 566.8223 | 27.78 | 3.904751 |
| FEFETIDTL | 1113.523 | 557.7696 | 38.31 | 2.217964 |
| EVTLGFVDLM(+15.99)RDDYIEKDR | 2329.126 | 583.2908 | 25.52 | 4.560224 |
| ISGLIYEETRGVLK | 1576.882 | 526.6371 | 17.76 | 4.498637 |
| SIDLLKDTLK | 1144.67 | 382.5654 | 19.51 | 3.92815 |
| KM(+15.99)KDTDSEAELLEAFK | 1869.903 | 624.3142 | 20.57 | 2.984812 |
| QLIVGVNKM(+15.99)DSDTAGYKEER | 2268.106 | 568.0391 | 8.95 | 4.053833 |
| LAEQVIYVAGDR | 1332.704 | 667.361 | 16.27 | 3.161809 |
| FVTFDGLAK | 996.528 | 499.2729 | 21.51 | 5.830532 |
| VAEAIDRDLGGYDK | 1520.747 | 507.9264 | 11.75 | 5.033037 |
| AVNVTGPN(+.98)GAPPEGAPR | 1603.795 | 802.9062 | 7.7 | 4.530311 |
| DYQELMNTK | 1140.512 | 571.2706 | 13.36 | 3.651428 |
| VLNFAVPDQLLVDR | 1597.883 | 799.9493 | 35.8 | 3.603157 |
| IGLFGGAGVGK | 974.5549 | 488.2861 | 19.93 | 8.062766 |

| | | | | |
|---|---|---|---|---|
| GIPAM(+15.99)LWETSLLDPEEGIR | 2142.067 | 715.0303 | 40.21 | 4.997188 |
| EQDFPGVVLR | 1158.603 | 580.311 | 21.31 | 4.574968 |
| QYN(+.98)EEN(+.98)GITPPARPPR | 1839.875 | 614.3008 | 9.2 | 4.144492 |
| LPISGIYK | 889.5273 | 445.7736 | 15.21 | 3.263962 |
| LVDILAQQYEIVAR | 1629.909 | 815.968 | 32.46 | 4.016139 |
| VTFPEASQIAEELLKTEFDTAR | 2494.259 | 832.4326 | 47.94 | 3.552058 |
| IQTFFGPGIVAK | 1276.718 | 639.361 | 26.05 | 3.301144 |
| DNIQ(+.98)GITKPAIR | 1325.73 | 442.9212 | 11.08 | 3.485204 |
| EISPEILN(+.98)AIR | 1254.682 | 628.3468 | 29.29 | 4.499454 |
| VDAIVFLVDAADRER | 1687.889 | 563.6407 | 36.28 | 3.927298 |
| EIYIDDLREEFVR | 1695.847 | 566.2898 | 30.7 | 6.181792 |
| TAIAVDTILNQK | 1285.724 | 643.8691 | 21.73 | 2.083183 |
| QGVDADINGLR | 1156.584 | 579.3008 | 12.63 | 7.56237 |
| VLLLPEDPNAVIIC(+57.02)VATGTGIAPFR | 2635.441 | 879.4843 | 48.68 | 3.300338 |
| DNIQGITKPAIR | 1324.746 | 442.591 | 9.64 | 3.485204 |
| LYFQ(+.98)PYSAEK | 1245.592 | 623.8052 | 18.57 | 3.489219 |
| TAIAVDTILNQ(+.98)K | 1286.708 | 644.352 | 20.96 | 2.083183 |
| DAVLLVFANK | 1088.623 | 545.319 | 29.75 | 2.735893 |
| IFVHPVADVIR | 1264.729 | 422.5876 | 16.53 | 3.272165 |
| DAEAWFNEK | 1108.483 | 555.2522 | 19.55 | 3.023328 |
| TVTAFDVVYALK | 1325.723 | 663.8688 | 34.79 | 2.995065 |
| KEQC(+57.02)LALLTQ(+.98)LR | 1472.802 | 491.943 | 23.83 | 4.474069 |
| EVVEGVDILIVR | 1339.771 | 670.8918 | 30.85 | 3.877383 |
| GIAELGIYPAVDPLDSTSR | 1973.011 | 987.5095 | 34.17 | 3.319146 |
| DIN(+.98)EVIAAGTAK | 1201.619 | 601.8208 | 19.06 | 4.669747 |
| GGLDFTKDDENVN(+.98)SQPFM(+15.99)R | 2185.959 | 729.6605 | 18.27 | 7.15936 |
| QQ(+.98)AEQLALALR | 1240.678 | 621.3444 | 20.82 | 3.8618 |
| GPWLEPLR | 966.5287 | 484.274 | 22.67 | 5.265771 |
| WAM(+15.99)LGVAGIVIPAELTR | 1811.997 | 907.0075 | 40.97 | 5.958201 |
| AIEDM(+15.99)LIM(+15.99)AAR | 1264.616 | 633.3189 | 18.61 | 3.723546 |
| RAQ(+.98)LGEIFEFDR | 1480.731 | 494.5868 | 27.42 | 5.677311 |
| ISTVQEAADLIAAQ(+.98)MDK | 1803.892 | 602.3055 | 43.78 | 3.345185 |
| VLEQELIPVQR | 1322.756 | 662.3861 | 19.55 | 2.381773 |
| AGAEIFGDDGIQ(+.98)YLGIPGLINAK | 2332.195 | 1167.104 | 44.73 | 5.450156 |
| YSEM(+15.99)VFPILAPDPAKN(+.98)K | 1935.965 | 646.3365 | 24.47 | 3.280432 |
| VPFELPK | 828.4745 | 415.2475 | 18.11 | 3.417281 |
| QSVEADINGLR | 1200.61 | 601.3178 | 15.04 | 4.544759 |
| VVAGPGDKPMIEVEFK | 1714.896 | 572.6429 | 19.38 | -0.05956 |
| LVFPEEVLPR | 1197.676 | 599.8467 | 28.8 | 2.970092 |
| DEVTVTLK | 903.4913 | 452.7554 | 11.08 | 2.224113 |
| EAAATAEALEAER | 1330.637 | 666.3284 | 10.4 | 3.849948 |
| IISLAPEVL | 953.5797 | 477.7986 | 35.67 | 2.534149 |
| EFVWATLK | 992.5331 | 497.2748 | 27.46 | 3.577465 |
| AIVISVIDNLVK | 1282.786 | 642.4014 | 40.1 | 4.342412 |

| | | | | |
|---|---|---|---|---|
| KEQ(+.98)DFPGVVLR | 1287.682 | 430.238 | 16.22 | 4.11599 |
| GAALN(+.98)AVQ(+.98)VAELLL | 1382.766 | 692.3897 | 53.61 | 4.916586 |
| LGWTVVPEQLR | 1296.719 | 649.3677 | 28.35 | 8.02127 |
| Q(-17.03)LIVGINKM(+15.99)DSDTAGYKQ(+.98)ER | 2265.095 | 567.2713 | 8.7 | 4.568232 |
| TLFYVPAPR | 1062.586 | 532.302 | 22.42 | 3.597046 |
| SSFAFAFYM(+15.99)DR | 1356.581 | 679.3005 | 30 | 4.333066 |
| AYDFVSQEIR | 1226.593 | 614.3152 | 19.03 | 5.646524 |
| EVTLGFVDLM(+15.99)RDDYIEK | 2057.998 | 687.0114 | 29.65 | 3.929279 |
| ALGVLSQGVWSR | 1271.699 | 636.8588 | 25.42 | 3.97957 |
| VAVLGASGYTGEEVVR | 1605.836 | 803.9232 | 17.75 | 4.800473 |
| LEQ(+.98)EQQFLIAK | 1346.708 | 674.36 | 18.09 | 4.089113 |
| YADIMAALK | 994.5157 | 498.2658 | 24.04 | 4.705672 |
| AGLQFPVGR | 943.5239 | 472.7722 | 17.37 | 5.874735 |
| GPWLEPLRGPN(+.98)GLDLNK | 1875.984 | 626.3356 | 28.66 | 4.046135 |
| GEEGNVQVAQAALLK | 1525.81 | 763.9156 | 17.46 | 4.00927 |
| VNDLLDFK | 962.5073 | 482.262 | 22.42 | 3.435457 |
| LSEQFVEAYR | 1240.609 | 621.317 | 16.27 | 2.90217 |
| QIVTLDM(+15.99)GLLVAGTK | 1573.875 | 787.943 | 27.62 | 1.998179 |
| DLGLPWVILGHSER | 1590.852 | 531.2913 | 37.2 | 3.426252 |
| VLEQ(+.98)ELIPVQ(+.98)R | 1324.724 | 663.3695 | 22.78 | 2.381773 |
| KGDLN(+.98)VGAVLILPEGFELAPPDR | 2420.295 | 807.7715 | 40.32 | 6.671945 |
| KGDLN(+.98)VGAVLILPEGFEIAPPDR | 2420.295 | 807.7715 | 40.32 | 6.671945 |
| IASGDVPETIEGK | 1314.667 | 658.3445 | 11.06 | 2.527053 |
| DIELVMTQ(+.98)AGVSR | 1418.708 | 710.3594 | 27.12 | 5.074851 |
| SN(+.98)YN(+.98)YEKPFLYLAR | 1778.851 | 593.9617 | 29.58 | 5.575304 |
| TIAEC(+57.02)LADELINAAK | 1630.824 | 544.6166 | 42.71 | 2.98891 |
| VADYVSQLR | 1049.551 | 525.786 | 12.91 | 5.212766 |
| GAGGAFVLVLYDEIKK | 1678.929 | 560.6525 | 37.03 | 2.451999 |
| TFAEEVN(+.98)EAFR | 1312.594 | 657.3105 | 17.35 | 3.033428 |
| GLALVAEKPLGSGVK | 1437.856 | 480.2947 | 14.58 | 4.470616 |
| VGKNN(+.98)IDELGSPDK | 1485.731 | 496.2549 | 8.38 | 3.773616 |
| EVTLGFVDLMR | 1278.664 | 640.3401 | 36.89 | 4.531163 |
| DFTAQ(+.98)IQTLDIPGEVK | 1774.899 | 888.4523 | 33.31 | 4.87734 |
| SIPDEDAGTEGAIFVFR | 1822.874 | 912.4536 | 32.21 | 3.027431 |
| AGIQLSDTFVK | 1177.634 | 589.8242 | 19.9 | 6.045974 |
| YPLVADLK | 917.5222 | 459.7705 | 17.65 | 2.554114 |
| Q(-17.03)FSAEEISSM(+15.99)VLSK | 1553.728 | 777.8737 | 34.87 | 2.535905 |
| WPYGEFEVPK | 1250.597 | 626.3073 | 26.97 | 2.758936 |
| LFTIALPR | 929.5698 | 465.7943 | 26.4 | 4.444546 |
| HPEIDVPNLEVIK | 1501.814 | 501.6155 | 24.88 | 3.655833 |
| SVPILPISGWM(+15.99)GDNLIK | 1854.991 | 619.3368 | 37.5 | 4.477592 |
| LADPLGFVK | 958.5487 | 480.2825 | 25.52 | 6.7761 |

| | | | | |
|---|---|---|---|---|
| LELQEVVDFLK | 1331.734 | 666.8737 | 42.35 | 4.270926 |
| YEDN(+.98)GDAVN(+.98)N(+.98)LVVLVQK | 1891.905 | 631.649 | 38.85 | 3.094416 |
| FN(+.98)LGTESLSAGVTWK | 1609.799 | 805.9069 | 27.66 | 1.427317 |
| TFAEEVNEAFR | 1311.61 | 656.8154 | 26.48 | 3.033428 |
| GFDIIYEAR | 1082.54 | 542.2786 | 23.48 | 5.941211 |
| RDEDGYYWILGR | 1541.726 | 514.9177 | 28.13 | 3.915063 |
| IDGPAGSLAAELR | 1268.672 | 635.3374 | 22.85 | 2.194342 |
| DINKQDVLLFIDNIFR | 1962.057 | 655.0269 | 49.24 | 2.772713 |
| NMQDM(+15.99)VEDYR | 1315.517 | 658.7698 | 10.81 | 3.85266 |
| QSVEADINGLRR | 1356.711 | 453.2454 | 12.09 | 4.268072 |
| IYNLM(+15.99)DQVDAYIPTPER | 2052.983 | 685.3443 | 29.76 | 3.711355 |
| DGVVVIIK | 841.5273 | 421.7748 | 16.04 | 5.602871 |
| REQAEQLALALR | 1396.779 | 466.604 | 14.82 | 4.401985 |
| KPDFDAYIDPQK | 1435.698 | 479.58 | 15.07 | 3.324166 |
| VQQLLQDFFN(+.98)GK | 1436.73 | 719.3697 | 34.08 | 3.976918 |
| LGAN(+.98)VASAQ(+.98)GPTGLGK | 1441.741 | 721.8816 | 12.5 | 7.971924 |
| IVSVETITGPK | 1142.655 | 572.3347 | 14.8 | 1.991136 |
| EVAAFAQFGSDLDAATQ(+.98)YLLNR | 2400.16 | 801.0656 | 50.14 | 2.847102 |
| IGVIESLLEK | 1099.649 | 550.8328 | 31.06 | 6.167008 |
| VLITTDLLAR | 1113.676 | 557.8464 | 25.94 | 4.192062 |
| AVDSLVPIGR | 1025.587 | 513.8011 | 17.13 | 3.631344 |
| TVLIMELIN(+.98)NVAK | 1457.816 | 729.9193 | 39.21 | 3.276097 |
| GLNVGSSLVEEDKLALAK | 1842.01 | 615.0134 | 21.8 | 4.887117 |
| VSVEAGSTYGWQK | 1410.678 | 706.3467 | 13.2 | 5.036921 |
| VPFAISPGSEQIR | 1399.746 | 700.8836 | 19.81 | 4.557735 |
| NLQDIIAILGM(+15.99)DELSEEDKM(+15.99) TVAR | 2735.336 | 684.8433 | 43.21 | 4.412734 |
| YTLEVDLK | 979.5226 | 490.7719 | 19.12 | 1.725241 |
| QATKDAGAIAGLEVLR | 1611.894 | 538.3073 | 19.37 | 4.221557 |
| ITGASTLPSDEVER | 1473.731 | 737.8748 | 11.24 | 2.40459 |
| QEYEQLIAK | 1120.576 | 561.2957 | 12.07 | 4.364527 |
| VLSVGDGIAR | 985.5556 | 493.7864 | 11.85 | 6.681897 |
| SDLEMQYETLQEELM(+15.99)ALKK | 2314.107 | 772.374 | 35.97 | 1.576727 |
| VLQ(+.98)VDEPALR | 1139.619 | 570.8189 | 15.18 | 3.840119 |
| ETEKPFLM(+15.99)AVEDVFSITGR | 2184.077 | 729.0316 | 46.82 | 1.919876 |
| VVVLGFFDK | 1022.58 | 512.2999 | 31.62 | 2.717435 |
| TM(+15.99)KPAAAQEYFAAI | 1526.744 | 764.3842 | 23.71 | 1.952421 |
| ISLVDAIVIR | 1097.681 | 549.8473 | 28.38 | 3.109851 |
| VVAWYDNEWGYSQ(+.98)R | 1772.779 | 887.4012 | 28.89 | 3.05539 |
| FKIDGYIYR | 1173.618 | 392.2169 | 18.18 | 4.113416 |
| IRLENEIQTYR | 1433.763 | 478.9312 | 13.01 | 3.038935 |
| LGAN(+.98)SLLDIVVFGR | 1473.819 | 737.9167 | 47.78 | 6.607616 |
| EFAEGILSK | 992.5178 | 497.2686 | 17.72 | 2.401805 |
| RVLQ(+.98)VDEPALR | 1295.72 | 432.9162 | 11.85 | 2.73176 |

| | | | | |
|---|---|---|---|---|
| IGELQAAAFK | 1046.576 | 524.2953 | 15.28 | 5.32073 |
| GVDILLAAIK | 1011.633 | 506.8249 | 32.79 | 4.289899 |
| GVDILLAALK | 1011.633 | 506.8249 | 32.79 | 4.193138 |
| GAYFGLYDTAK | 1204.576 | 603.3019 | 20.85 | 5.224535 |
| IGGLEVLR | 855.5178 | 428.7683 | 17.34 | 7.000569 |
| SAVDEAIEM(+15.99)LK | 1220.596 | 611.3046 | 22.39 | 4.417288 |
| AFM(+15.99)QADLQAELIELLEK | 1977.013 | 989.5086 | 47.4 | 3.255782 |
| DVEDALVLIK | 1113.628 | 557.8214 | 30.49 | 2.623573 |
| TGFVLDGFPR | 1107.571 | 554.7925 | 28.23 | 9.315999 |
| FAGPIAIIR | 956.5807 | 479.2968 | 23.55 | 3.701028 |
| GELVSLASIFR | 1190.666 | 596.3403 | 38.13 | 4.211838 |
| AGAQIFQEGGLDYLGN(+.98) | 1652.768 | 827.3931 | 39.57 | 6.067209 |
| DSTLIM(+15.99)QLLR | 1204.649 | 603.3298 | 35.43 | 5.185824 |
| LYFEPLTVEDVLNVIEK | 2020.077 | 1011.044 | 50.44 | 3.618698 |
| DGLIEIFEK | 1062.56 | 532.2922 | 33.47 | 5.994914 |
| KEQIFEM(+15.99)PTGGAAIM(+15.99)R | 1809.875 | 604.3065 | 11.2 | 4.751171 |
| DLDLPQ(+.98)GVR | 1012.519 | 507.2694 | 17.34 | 5.709387 |
| QDVLLFIDNIFR | 1491.809 | 746.9098 | 49.35 | 3.917743 |
| MTLDDFR | 896.4062 | 449.2133 | 18.35 | 2.302494 |
| EGAAALADLLR | 1098.603 | 550.3094 | 29.01 | 7.30722 |
| EFDAEILR | 991.4974 | 496.7592 | 23.02 | 5.278389 |
| IAGLDVLR | 855.5178 | 428.7684 | 20.49 | 4.225432 |
| FLSQPFFVAEVFTG | 1587.797 | 794.9064 | 49.82 | 0.632967 |
| FLEYLEM(+15.99)GR | 1172.554 | 587.2877 | 21.84 | 5.920323 |
| EDFEGIFTAM(+15.99)DGLPVTIR | 2025.972 | 676.338 | 38.64 | 3.704175 |
| IPAVQELVEK | 1124.644 | 563.3309 | 18.4 | 1.953544 |
| ETRLIESPAPGIISRR | 1794.011 | 449.5129 | 11.88 | 1.144952 |
| EAEKRIDADLDAYIAK | 1819.932 | 455.9936 | 16.18 | 3.508442 |
| VLQVDEPALR | 1138.635 | 570.319 | 14.17 | 3.840119 |
| GLSADPETFAR | 1162.562 | 582.2935 | 12.21 | 5.624408 |
| AVALVLPK | 809.5374 | 405.779 | 16.43 | 1.854481 |
| VVGFDPVR | 887.4865 | 444.7548 | 13.98 | 3.266639 |
| SFFDGFTDK | 1062.466 | 532.2421 | 26.58 | 2.724816 |
| ATLSYFQAVK | 1126.602 | 564.3104 | 18.96 | 2.162645 |
| ILSEEFGWDK | 1222.587 | 612.3088 | 25.45 | 6.292409 |
| GM(+15.99)EVVDSGKPLNVPVGQATLGR | 2239.163 | 747.3863 | 16.21 | 5.224847 |
| TVLIM(+15.99)ELINN(+.98)VAK | 1473.811 | 492.2838 | 31.94 | 3.276097 |
| YAMLVDDGTVK | 1210.59 | 606.3069 | 15.32 | 6.482394 |
| LLLIGDSGVGK | 1070.634 | 536.3234 | 21.48 | 5.800573 |
| LIESPAPGIISRR | 1407.82 | 470.2835 | 12.65 | 0.86394 |
| LLAALGGN(+.98)AAPAEADIK | 1594.857 | 798.4342 | 21.6 | 2.953772 |
| AGAQIFADGGLN(+.98)YLGN | 1580.747 | 791.3909 | 34.97 | 6.067209 |
| DINKQ(+.98)DVLLFIDNIFR | 1963.041 | 655.3537 | 49.92 | 2.772713 |
| LNQIPLFIK | 1084.664 | 543.3387 | 28.16 | 5.554716 |

| | | | | |
|---|---|---|---|---|
| GTAGYFVEPTVFR | 1442.719 | 722.3667 | 27.05 | 1.898888 |
| DM(+15.99)IGILEGAIR | 1202.633 | 602.3264 | 27.82 | 5.161221 |
| YAM(+15.99)LVDDGTVK | 1226.585 | 614.3003 | 10.78 | 6.482394 |
| KEQDFPGVVLR | 1286.698 | 429.9128 | 15.73 | 4.11599 |
| ALEEANADLEVK | 1300.651 | 651.3365 | 11.69 | 3.521221 |
| YADIM(+15.99)AALK | 1010.511 | 506.2685 | 13.5 | 4.705672 |
| LVLPGELAK | 938.58 | 470.2993 | 18.42 | 3.321967 |
| VTLVYGQM(+15.99)N(+.98)EPPGAR | 1647.793 | 824.9133 | 13.34 | 2.617379 |
| IGDTVELVGLRDTK | 1514.83 | 505.954 | 16.78 | 5.7574 |
| VAEFAFDYAR | 1187.561 | 594.7896 | 22.71 | 5.828962 |
| YPLVADLKK | 1045.617 | 523.8161 | 11.75 | 2.437995 |
| DLEKLSIEEVM(+15.99)LK | 1561.827 | 521.6176 | 23.69 | 2.522041 |
| GFGILDIK | 861.496 | 431.7548 | 27.88 | 3.310065 |
| NGVANFGEAVWFK | 1437.704 | 719.8643 | 36.71 | 5.910272 |
| AYWTEGRN(+.98)ELR | 1394.658 | 465.8973 | 11.96 | 5.149186 |
| FTQAN(+.98)SEVSALLGR | 1492.752 | 747.3858 | 27.05 | 3.942627 |
| YEDNGDAVN(+.98)N(+.98)LVVLVQK | 1890.921 | 631.3234 | 32.09 | 3.094416 |
| VYFDITIDDKPAGR | 1608.815 | 537.287 | 22.39 | 4.397501 |
| LGELLDTVASLR | 1285.724 | 643.8702 | 32.8 | 7.152255 |
| LQEVIDEIR | 1113.603 | 557.8119 | 16.33 | 4.506228 |
| VVAAYLLAALGGNAAPAEADIK | 2097.147 | 700.0569 | 40.1 | 1.687362 |
| ATAELKN(+.98)GEVLLLEN(+.98)VR | 1870.005 | 624.3433 | 27.85 | 3.916233 |
| GDLNVGAVLILPEGFELAPPDR | 2291.216 | 764.7475 | 45.14 | 1.943136 |
| GDLNVGAVLILPEGFEIAPPDR | 2291.216 | 764.7475 | 45.14 | 1.943136 |
| LEGEREVTLGFVDLM(+15.99)R | 1878.951 | 627.3263 | 25.84 | 4.031899 |
| EILLQPPSGDELPAR | 1633.867 | 817.9459 | 23.67 | 3.854829 |
| TGEVLPERLPEK | 1366.746 | 456.5918 | 10.34 | 5.298619 |
| ELEFYVK | 926.4749 | 464.2468 | 18.81 | 5.473324 |
| QLSADVGIPR | 1054.577 | 528.2961 | 13.42 | 5.903505 |
| QLIVGVN(+.98)KM(+15.99)DSDTAGYKEER | 2269.09 | 568.2863 | 10.14 | 4.053833 |
| EQ(+.98)AEQLALALR | 1241.662 | 621.8427 | 22.39 | 3.919039 |
| QVGVPNIVVFLNK | 1425.834 | 713.9278 | 34.69 | 2.141885 |
| ISISTSGGSFR | 1110.567 | 556.2933 | 12.87 | 3.948274 |
| LKDEAVLLALAQK | 1410.845 | 471.2897 | 23.3 | 3.489814 |
| VYINQGM(+15.99)GVGAFR | 1426.703 | 714.3588 | 15.51 | 4.424862 |
| AADAVVAAIDQAALAIFLAQK | 2069.152 | 690.7237 | 52.75 | 2.842295 |
| FEELNM(+15.99)DLFR | 1328.607 | 665.3149 | 25.91 | 4.613312 |
| LLNLLGVK | 868.5746 | 435.2964 | 26.36 | 4.303997 |
| SAFIDFFR | 1001.497 | 501.7547 | 35.32 | 5.511563 |
| GIYPAVDPLDSTSR | 1489.741 | 745.8846 | 21.81 | 3.613032 |
| VNKVQEQFGN(+.98)AM(+15.99)GADDFM(+15.99)QR | 2317.01 | 773.3484 | 11.52 | 3.860506 |
| VLDELTLAR | 1028.587 | 515.2961 | 18.91 | 4.925371 |
| IAGLEVLR | 869.5334 | 435.7755 | 19.44 | 4.473435 |

| | | | | |
|---|---|---|---|---|
| YPIYVGGN(+.98)R | 1038.513 | 520.2693 | 12.91 | 4.807981 |
| VRTIPLEVM(+15.99)VK | 1299.758 | 434.2635 | 14.65 | 3.875368 |
| Q(-17.03)NLEPLFEQYINNLR | 1872.937 | 937.4764 | 49.79 | 5.49873 |
| VSASQIVADLSK | 1216.666 | 609.3317 | 27.6 | 2.250846 |
| IVSKFEEIAAEQR | 1518.804 | 507.2741 | 11.89 | 1.577192 |
| VYIN(+.98)Q(+.98)GM(+15.99)GVGAFR | 1428.671 | 715.3436 | 17.95 | 4.424862 |
| AYWTEGRNELR | 1393.674 | 465.5688 | 10.69 | 5.149186 |
| DIN(+.98)KQDVLLFIDNIFR | 1963.041 | 655.3553 | 50.17 | 2.772713 |
| TVLIMELINNIAK | 1470.848 | 736.4315 | 48.21 | 3.547136 |
| TGDIVLVGLR | 1041.618 | 521.818 | 22.67 | 6.93384 |
| DN(+.98)GLLLHIHR | 1187.641 | 396.8941 | 14.26 | 3.308738 |
| VLEQLTGQQPVFGK | 1542.841 | 772.4413 | 21 | 3.334917 |
| EFEFAFK | 916.433 | 459.226 | 26.26 | 2.873072 |
| YKELQDIIAILGLDELSEEDRLIVAR | 3013.634 | 1005.552 | 47.72 | 4.551475 |
| VVFDFVEK | 981.5171 | 491.7683 | 22.81 | 2.756347 |
| TRLEQEIATYR | 1378.721 | 460.5855 | 12.8 | 2.578021 |
| QSLLQPLNVEIDPEIQK | 1963.063 | 655.3632 | 32.91 | 3.125521 |
| KEEKVEEEEDEDM(+15.99)GFSLFD | 2319.958 | 774.3337 | 24.09 | 2.522927 |
| AFPDAYIR | 951.4814 | 476.7506 | 17.27 | 5.365223 |
| AGAQIFQ(+.98)EGGLDYLGN | 1652.768 | 827.3961 | 39.14 | 6.067209 |
| VVLADIAVGK | 983.6015 | 492.8096 | 18.74 | 2.073574 |
| GQLQEALEFAQEYLAPR | 1961.985 | 655.0048 | 47.46 | 3.240412 |
| VAAASVLDVQEATDKKGK | 1828.989 | 458.2549 | 9.68 | 3.912469 |
| IVEVLLEK | 941.5797 | 471.8 | 17.69 | 2.790797 |
| AKDFETFLEPLFAAFK | 1872.966 | 625.3292 | 48.42 | 3.727224 |
| VYVSNLAWR | 1106.587 | 554.2958 | 21.51 | 2.634664 |
| ANLVVFPR | 914.5338 | 458.2779 | 18.77 | 3.946264 |
| SILGDGC(+57.02)IVEPDAR | 1500.724 | 751.3743 | 19.67 | 1.852066 |
| EYSSEVILAK | 1137.592 | 569.804 | 12.91 | 3.790929 |
| FDIIYEAR | 1025.518 | 513.7692 | 20.63 | 4.027463 |
| VTIM(+15.99)PKDIQLAR | 1399.786 | 467.6042 | 13.18 | 3.794464 |
| VFPN(+.98)GEVQYLHPK | 1527.772 | 510.2735 | 16.5 | 2.294477 |
| AVITVPAYFN(+.98)DSQR | 1580.783 | 791.4026 | 26.45 | 2.206031 |
| VNIFPIR | 857.5123 | 429.7639 | 22 | 4.606834 |
| VLYLPGGLLAR | 1170.712 | 586.3612 | 31.13 | 5.481946 |
| QVQVSGFLVNRPSSVR | 1771.969 | 591.6651 | 16.44 | 3.808595 |
| AGLQ(+.98)FPVGR | 944.5079 | 473.2638 | 18.6 | 5.874735 |
| DFTAQIQ(+.98)TLDIPGEVK | 1774.899 | 888.4579 | 33.03 | 4.87734 |
| GVLLVGPPGTGK | 1093.65 | 547.8344 | 16.15 | 5.365366 |
| IVPVTLELGGK | 1124.68 | 563.3513 | 19.3 | 3.166793 |
| LGIN(+.98)GFGR | 833.4395 | 417.7303 | 16.78 | 9.153717 |
| ALGLPIERPK | 1092.666 | 365.2323 | 12.83 | 3.893796 |
| E(-18.01)IKFEFETIDTL | 1465.734 | 733.874 | 40.03 | 0.976619 |
| VADYVSQ(+.98)LR | 1050.535 | 526.2778 | 14.51 | 5.212766 |

| | | | | |
|---|---|---|---|---|
| DIN(+.98)KQ(+.98)DVLLFIDNIFR | 1964.025 | 655.6825 | 50.63 | 2.772713 |
| ASTLQDLLEK | 1116.603 | 559.3079 | 24.36 | 3.702676 |
| WELLQQVNTSTR | 1473.758 | 492.2597 | 26.12 | 3.665902 |
| TAGLSADPETFAR | 1334.647 | 668.3397 | 14.91 | 4.291674 |
| LYVTGQ(+.98)FLGYK | 1288.67 | 645.3419 | 26.83 | 2.464068 |
| DYQELMNVK | 1138.533 | 570.2762 | 17.48 | 4.366107 |
| VFEIGPVFR | 1062.586 | 532.3008 | 28.87 | 2.747289 |
| FGAQLFR | 837.4497 | 419.7352 | 18.11 | 6.255172 |
| SLLDIVVFGR | 1117.65 | 559.8327 | 42.28 | 4.335317 |
| TVIAFVDDPTGYK | 1424.719 | 713.3657 | 24.13 | 0.599964 |
| IINEPTAASLAYGLDKK | 1802.978 | 601.9983 | 15.64 | 1.235513 |
| IPDWFLNR | 1059.55 | 530.7814 | 29.68 | 4.285133 |
| WN(+.98)PSKEQDFPGVVLR | 1771.889 | 591.6418 | 23.31 | 4.253433 |
| LIFPYVDLK | 1106.638 | 554.3281 | 33.82 | 2.279278 |
| AAAALADTKAKLDAVIAK | 1740.014 | 436.0157 | 12.51 | 2.545651 |
| TTLTAAITM(+15.99)ALAAR | 1419.776 | 474.2673 | 36.42 | 2.495911 |
| LGDEEAGLIGGN(+.98)QLR | 1541.769 | 771.8904 | 21.99 | 8.707296 |
| DFVFPM(+15.99)FR | 1073.5 | 537.7583 | 32.9 | 3.510474 |
| QNLEPLFEQYINNLR | 1889.964 | 630.9958 | 42.64 | 5.49873 |
| AVIAFLDELVK | 1216.707 | 609.3608 | 41.49 | 3.341127 |
| IGLIGDLAN(+.98)GR | 1098.603 | 550.3014 | 23.76 | 7.129676 |
| TAELAIN(+.98)AIR | 1071.592 | 536.8054 | 18.32 | 5.050767 |
| LN(+.98)QIPLFIK | 1085.648 | 543.8318 | 29.96 | 5.554716 |
| YAVVSAIAASALPALVM(+15.99)AR | 1889.044 | 630.6887 | 40.38 | 4.56278 |
| TSQNSELNNMQ(+.98)DLVEDYKK | 2256.022 | 753.0206 | 25 | 2.5751 |
| ILM(+15.99)VGLDAAGK | 1102.606 | 552.308 | 15.66 | 3.962946 |
| FVDLGADSLDTVEIM(+15.99)M(+15.99)ALEEK | 2357.102 | 1179.564 | 38.87 | 3.099348 |
| DSVLLPEVK | 998.5648 | 500.2907 | 19.05 | 2.549355 |
| GAALN(+.98)AVQVAELLL | 1381.782 | 691.8975 | 51.64 | 4.916586 |
| LALQELVDK | 1027.591 | 514.8057 | 21.86 | 2.167461 |
| VPFAISPGSEQ(+.98)IR | 1400.73 | 701.3635 | 21.03 | 4.557735 |
| IM(+15.99)YALTAIK | 1038.578 | 520.2951 | 17.13 | 3.172557 |
| GGLDFTKDDEN(+.98)VN | 1423.61 | 712.8136 | 15.19 | 6.73869 |
| YFDFEFTAK | 1166.528 | 584.2742 | 29.15 | 4.554449 |
| AVQLVILPR | 1007.649 | 504.8337 | 23.4 | 3.159082 |
| IFIEFLER | 1065.586 | 533.8004 | 34.11 | 3.782676 |
| VFLENVVR | 974.5549 | 488.2876 | 19.19 | 4.298178 |
| GFDPLGLSR | 960.5028 | 481.2614 | 24.43 | 6.501442 |
| ELLSFYK | 898.48 | 450.2503 | 20.42 | 3.06093 |
| VN(+.98)DLLDFK | 963.4913 | 482.7529 | 24.64 | 3.435457 |
| EDIVN(+.98)VIK | 929.507 | 465.764 | 22.7 | 3.582394 |
| A(+42.01)AVAEELRK | 1027.566 | 514.7931 | 14.65 | 2.266066 |
| LLN(+.98)LLGVK | 869.5586 | 435.7882 | 28.27 | 4.303997 |

| | | | | |
|---|---|---|---|---|
| IFN(+.98)VLGEPVDN(+.98)LGPVN | 1697.851 | 849.9336 | 20.09 | 4.410864 |
| VVILLSGR | 855.5541 | 428.7881 | 16.6 | 3.339176 |
| AGASIFN(+.98)EDGLNYLGN | 1654.747 | 828.3892 | 39.11 | 6.03802 |
| VLEWFR | 848.4545 | 425.2354 | 26.08 | 5.857105 |
| M(+15.99)EFPDPVIK | 1090.537 | 546.2723 | 16.68 | 3.441903 |
| LNAEQALEM(+15.99)AFYVASR | 1827.882 | 610.3053 | 44.28 | 3.028035 |
| GVDLEQLLDM(+15.99)STDELVELFPAR | 2505.231 | 836.0873 | 53.05 | 5.079449 |
| AEELLEQLR | 1099.587 | 550.8052 | 20.07 | 5.399862 |
| GLVVPVLR | 851.5593 | 426.7897 | 21.79 | 4.495641 |
| LN(+.98)DVEDVER | 1088.499 | 545.2657 | 9.79 | 5.280097 |
| SM(+15.99)AILN(+.98)SFIVDQFEK | 1757.855 | 879.9428 | 39.08 | 4.101922 |
| IPSAVGYQPTLATDLGQLQER | 2256.175 | 753.0625 | 30.29 | 3.29774 |
| YPVVVRFEK | 1135.639 | 379.5575 | 12.34 | 3.788755 |
| KIGDTLEEFLLEATPDPK | 2015.046 | 672.6815 | 36.85 | 1.126526 |
| VLEQ(+.98)ELIPVQR | 1323.74 | 662.8674 | 21.31 | 2.381773 |
| FDIDAN(+.98)GILSVTATDKGTGK | 2023.011 | 675.3553 | 25.56 | 4.402523 |
| TAIAVDTILN(+.98)QK | 1286.708 | 644.3494 | 18.12 | 2.083183 |
| GQLPQQPVVVR | 1219.704 | 610.8594 | 10.74 | 3.483201 |
| LTVAIEDPR | 1012.555 | 507.2852 | 13.21 | 2.415986 |
| Q(-17.03)SVEADINGLRR | 1339.684 | 670.8515 | 18.75 | 4.268072 |
| MSIADIPR | 901.4691 | 451.7457 | 16.95 | 3.088563 |
| YGVELIGAK | 948.528 | 475.2749 | 15.8 | 5.754656 |
| QGQFQPM(+15.99)PIENQIVVLYAATR | 2418.237 | 807.0841 | 36.26 | 6.690697 |
| ADLN(+.98)VPLDGDLK | 1269.645 | 635.8294 | 22.53 | 3.52292 |
| QFADVLAAVR | 1088.598 | 545.3071 | 26.12 | 3.710027 |
| SKEQDFPGVVLR | 1373.73 | 458.925 | 14.61 | 3.233406 |
| LAM(+15.99)VSFLGF | 999.5099 | 500.7608 | 44.17 | 5.720883 |
| VAVLGAAGGIGQPLSLLM(+15.99)K | 1810.039 | 604.345 | 34.82 | 3.397347 |
| VLTPAEVADYLAEVE | 1617.814 | 809.9134 | 47.32 | 2.580738 |
| Q(+.98)RAQARGGTAAAAAAK | 1498.796 | 500.6027 | 10.24 | 2.705767 |
| PVLIGEPGVGK | 1064.623 | 533.319 | 13.78 | 4.361604 |
| ISGLIYEETR | 1179.614 | 590.8173 | 15.36 | 3.121454 |
| AIDLIDEAGSR | 1158.588 | 580.2949 | 16.33 | 3.979281 |
| AIDLLDEAGSR | 1158.588 | 580.2949 | 16.33 | 3.979281 |
| RVLELSLEEAR | 1313.73 | 438.9197 | 15.21 | 4.021122 |
| EQLLLEIK | 984.5855 | 493.3007 | 23.37 | 3.549154 |
| LWELLK | 800.4796 | 401.2494 | 26.89 | 5.482051 |
| ALLELEEELRK | 1341.75 | 448.2585 | 22.49 | 2.626992 |
| WEAVLTELK | 1087.591 | 544.8084 | 25.02 | 3.224031 |
| SLN(+.98)LTDITK | 1004.539 | 503.2787 | 15.91 | 3.709303 |
| LPM(+15.99)FGC(+57.02)TDGGQVIR | 1565.733 | 783.876 | 19.67 | 4.761224 |
| DNVALPGIAAFFK | 1361.734 | 681.8746 | 44.17 | 3.426364 |
| LALQELVDKTQQ(+.98)FVEGTVR | 2174.158 | 725.725 | 45.46 | 5.717316 |
| QM(+15.99)VLDLLQTLEK | 1445.78 | 723.8918 | 36.82 | 3.400081 |

| | | | | |
|---|---|---|---|---|
| EGLFEIGR | 919.4763 | 460.748 | 22.67 | 8.059816 |
| EGLFELGR | 919.4763 | 460.748 | 22.67 | 8.333114 |
| LLFEALK | 832.5058 | 417.2619 | 24.18 | 4.855168 |
| DLAVQTVLNVGSR | 1370.752 | 457.9254 | 21.4 | 2.859214 |
| SPAEGAYSEGFIR | 1382.647 | 692.3398 | 15.5 | 5.933137 |
| FTIEEIR | 906.4811 | 454.2501 | 17.41 | 3.646572 |
| VLNLEEGGAFTVSGAEDILAALS | 2275.158 | 1138.586 | 51.69 | 3.118395 |
| VEPYVAYGYPNLK | 1511.766 | 756.8928 | 21.67 | 3.44017 |
| TSQNSELNNM(+15.99)QDLVEDYKK | 2271.033 | 758.0211 | 11.31 | 2.5751 |
| FGLYEYFK | 1065.517 | 533.7653 | 31.06 | 6.950443 |
| LPWGAM(+15.99)NIDLVIEGTGVFIDEAGAGK | 2688.347 | 897.1262 | 50.86 | 4.436161 |
| AGIQ(+.98)LSDTFVK | 1178.618 | 590.3085 | 22.29 | 6.045974 |
| GEFPGDYGWDTAGLSADPQTFAR | 2457.087 | 820.0232 | 33.81 | 5.650221 |
| SIGINEFGASAPGPLLYEK | 1962.01 | 982.0042 | 32.89 | 3.128158 |
| KGDLN(+.98)VGAVLILPEGFELAPPDRIPEEM(+15.99)K | 3163.648 | 791.9232 | 37.04 | 7.172476 |
| KGDLN(+.98)VGAVLILPEGFEIAPPDRIPEEM(+15.99)K | 3163.648 | 791.9232 | 37.04 | 7.172476 |
| AYWTEGRN(+.98)ELRR | 1550.759 | 388.7003 | 8.94 | 3.823191 |
| ADILDN(+.98)ALLRPGR | 1423.778 | 475.6003 | 23.93 | 3.930987 |
| TVFC(+57.02)IALK | 950.5259 | 476.2709 | 22.46 | 2.177559 |
| LATAQ(+.98)AGLAEAQAK | 1342.709 | 672.3666 | 15.75 | 4.014241 |
| EGGEPIPEGLLWLLM(+15.99)TGEM(+15.99)PTVDQAK | 2842.377 | 948.4745 | 48.01 | 6.271215 |
| QFPTVGFEK | 1051.534 | 526.7772 | 18.18 | 5.551895 |
| A(+42.01)AVAEELR | 899.4712 | 450.7443 | 21.02 | 4.169492 |
| ITVTSEIPM(+15.99)SK | 1220.632 | 611.3225 | 10.46 | 1.614229 |
| RISGLIYEETR | 1335.715 | 446.2482 | 12.2 | 3.492581 |
| AELLQYFR | 1038.55 | 520.284 | 27.14 | 4.934594 |
| VLDITDDDLGN(+.98)LITAGLR | 1913.995 | 958.0082 | 45.26 | 5.160092 |
| EWELSFR | 965.4606 | 483.7397 | 26.61 | 4.276404 |
| LDLFDDR | 892.429 | 447.2246 | 21.23 | 2.590032 |
| Q(-17.03)M(+15.99)VLDLLQTLEK | 1428.753 | 715.3823 | 48.17 | 3.400081 |
| Q(-17.03)IIEGGEFFVLK | 1361.723 | 681.8671 | 44.71 | 2.940485 |
| DELTLEGIK | 1016.539 | 509.2797 | 19.23 | 3.665898 |
| SLVDELRPELPER | 1551.826 | 518.2823 | 24.78 | 3.414682 |
| FWDFR | 769.3547 | 385.6863 | 24.35 | 5.286684 |
| AAVEEVRAQIAALAPA | 1578.873 | 790.4423 | 20.83 | 1.201645 |
| LAGGVAVIK | 826.5276 | 414.2717 | 10.03 | 2.547224 |
| VATVSLPR | 841.5021 | 421.7596 | 10.62 | 4.215673 |
| INIDEIGR | 928.4977 | 465.2605 | 14.44 | 5.168341 |
| LIPDDDDEELVEK | 1528.714 | 765.3652 | 15.2 | 2.818715 |
| YVVLF | 639.3632 | 640.3691 | 35.53 | 3.326262 |

| | | | | |
|---|---|---|---|---|
| EFPNVLTLR | 1087.603 | 544.8099 | 28.97 | 3.849946 |
| LAVGDKLPTDVK | 1254.718 | 419.2485 | 11.15 | 2.189824 |
| DFVFPMFR | 1057.506 | 529.7596 | 40.24 | 3.510474 |
| EVFDFPALR | 1092.56 | 547.2861 | 30.64 | 3.767774 |
| N(+.98)LQDIIAILGM(+15.99)DELSEEDKM(+15.99)TVAR | 2736.32 | 913.1182 | 45.9 | 4.412734 |
| FIEAFAAYLK | 1171.628 | 586.8267 | 33.83 | 3.800309 |
| VN(+.98)YAGVSTN(+.98)NYALDEVEEVKA | 2286.054 | 763.0262 | 29.2 | 1.759802 |
| DLGEAIEAFLK | 1204.634 | 603.3243 | 47.07 | 3.908448 |
| FLVRNM(+15.99)VDASALR | 1506.798 | 503.2754 | 11.36 | 2.985447 |
| WAM(+15.99)AAVAGILFTEILGK | 1805.975 | 602.9988 | 48.88 | 4.228942 |
| IM(+15.99)NVIGEPVDEK | 1358.675 | 680.348 | 11.76 | 1.995767 |
| TAIAIDAIINQ(+.98)K | 1270.713 | 636.3608 | 24.12 | 1.493575 |
| TVAQALEELEGR | 1314.678 | 658.3425 | 30.04 | 2.544612 |
| ISTVQ(+.98)EAADLIAAQ(+.98)M(+15.99)DK | 1820.871 | 607.9688 | 33.87 | 3.345185 |
| AAAAAAAAASAAAAKQAADQ(+.98)AAAK | 2012.029 | 671.6837 | 5.32 | 2.807077 |
| IFNVLGEPVDN(+.98)LGPVN | 1696.867 | 849.4438 | 18.86 | 4.410864 |
| VM(+15.99)GVTTLDVVR | 1204.649 | 603.3239 | 15.61 | 3.872423 |
| EVFDDYLR | 1055.492 | 528.7567 | 21.05 | 5.761082 |
| QLNEGQ(+.98)AGDNVGLLIR | 1696.874 | 849.4434 | 26.81 | 5.232613 |
| DIIPLLEQVTR | 1295.745 | 648.8798 | 38 | 2.602901 |
| GPPLFELLDEIESAAR | 1755.904 | 586.3099 | 51.1 | 4.494698 |
| DLTEDLLAK | 1016.539 | 509.2776 | 21.86 | 4.440346 |
| LLINVPDELLIDR | 1521.877 | 761.9478 | 38.68 | 3.157852 |
| DSLYPGGFFDPLGLADDPDTLAELK | 2665.28 | 889.4266 | 49.04 | 2.734123 |
| ATFDFRPGM(+15.99)IGK | 1354.67 | 452.5676 | 16.22 | 3.809258 |
| LFEGEALLR | 1046.576 | 524.2953 | 23.55 | 3.972259 |
| VGVLN(+.98)VPDWAEAGK | 1454.741 | 728.3735 | 30.22 | 6.456392 |
| GFAHVQFEELEGAAK | 1631.794 | 544.9403 | 19.94 | 4.492284 |
| GLTLIFVETK | 1119.654 | 560.8336 | 30.7 | 3.392461 |
| STQLELHIVTK | 1267.714 | 423.5823 | 12.65 | 1.692475 |
| STTTGRLLFELGGIPER | 1845.995 | 616.3336 | 28.03 | 2.046631 |
| TGDVM(+15.99)ILKSDATLK | 1506.796 | 503.2748 | 12.62 | 6.459877 |
| LADALLRPR | 1023.619 | 512.8127 | 21.16 | 4.885358 |
| DVVIVGVR | 855.5178 | 428.7713 | 14.47 | 3.034261 |
| YALPIDC(+57.02)KPIR | 1344.722 | 449.2501 | 15.07 | 4.40461 |
| VASLIEEVAR | 1085.608 | 543.8127 | 20.85 | 5.260151 |
| IVFVDN(+.98)EDFLKELEQK | 1965.993 | 656.3271 | 37.56 | 2.303437 |
| VLQDYRNLQDIIAILGM(+15.99)DELSEEDKM(+15.99)TVAR | 3509.738 | 878.4498 | 44.83 | 3.876194 |
| STN(+.98)ALTDEAQALLK | 1474.752 | 738.3862 | 30.4 | 2.693701 |
| DVLGVVSIDK | 1043.586 | 522.7986 | 23.09 | 1.134097 |
| EAVGTWN(+.98)SFTVV | 1309.619 | 655.8162 | 36.75 | 2.402072 |
| DVKQYN(+.98)EEN(+.98)GITPPARPPR | 2182.065 | 546.5233 | 9.22 | 2.068624 |

| | | | | |
|---|---|---|---|---|
| KSPLTKPADAM(+15.99)ER | 1458.75 | 487.2529 | 13.35 | 4.925089 |
| GLVTEVLR | 885.5283 | 443.7716 | 17.86 | 5.112097 |
| LLAELEGK | 871.5015 | 436.762 | 13.46 | 3.4709 |
| VLPWFTKEEIEK | 1517.813 | 506.9503 | 24.21 | 4.415581 |
| LILWDLR | 927.5541 | 464.7864 | 32.86 | 4.467812 |
| YLPFLQK | 907.5167 | 454.7675 | 24.21 | 4.294557 |
| VLPWFTK | 889.5062 | 445.7603 | 27.32 | 5.10622 |
| SISISVAR | 831.4814 | 416.75 | 11.01 | 4.141661 |
| VN(+.98)IFPIR | 858.4963 | 430.2503 | 23.69 | 4.606834 |
| LN(+.98)DVEDVERELPGELKK | 1983.016 | 662.0054 | 26.48 | 4.494859 |
| LGDEEAGLIGGNQ(+.98)LR | 1541.769 | 771.8938 | 21.74 | 8.707296 |
| VVDLLAPY | 888.4956 | 445.2553 | 33.54 | 3.468952 |
| ELIEDLTVLR | 1199.676 | 600.8416 | 30.88 | 3.953088 |
| KDDIELEPRFN(+.98)LGTESLSAGVTWK | 2705.355 | 677.3474 | 29.54 | 2.437032 |
| EGLLAYLR | 933.5283 | 467.7749 | 26.65 | 8.255118 |
| Q(-17.03)AGFDIIYEARDLDLPQ(+.98)GVR | 2259.117 | 754.044 | 40.71 | 4.623152 |
| M(+42.01)(+15.99)KN(+.98)DEGEIVDLYIPR | 1849.877 | 617.6369 | 31.66 | 4.646898 |
| VEIPAFIPRPDLIDQLVR | 2090.189 | 697.7372 | 43.04 | 4.243194 |
| ILGLLLDM(+15.99)TR | 1159.664 | 580.8414 | 28.45 | 4.35454 |
| AFLVGLR | 774.4752 | 388.2451 | 21.16 | 3.200763 |
| ALLQDIAIVTGAEFVAK | 1757.993 | 587.0084 | 45.64 | 3.091891 |
| AEVLGDLVER | 1099.587 | 550.8023 | 18.92 | 3.872045 |
| L(+42.01)TYYTPDYQPKDTDILAAFR | 2432.19 | 811.7375 | 40.68 | 3.180763 |
| TDN(+.98)ANILLQIDNAR | 1570.795 | 786.3933 | 39.35 | 3.468261 |
| LSADPETFAR | 1105.54 | 553.7839 | 12.77 | 4.189782 |
| WVLPLN(+.98)GR | 954.5287 | 478.2725 | 26.86 | 3.703407 |
| E(-18.01)SMELYQ(+.98)DLFVPK | 1580.743 | 791.391 | 33.74 | 2.960097 |
| LTTREGQLVVEAEK | 1571.852 | 524.957 | 9.65 | 1.833367 |
| IFNVLGEPVDNLGPVN(+.98) | 1696.867 | 849.4421 | 18.62 | 4.410864 |
| LAAVLFPTGETLGDVR | 1657.904 | 829.9496 | 31.75 | 5.848414 |
| GFPFEVPQ(+.98)EYANLPQLK | 1976.988 | 989.5074 | 38.44 | 4.329332 |
| LAGDYGFDPLGLGVDSDRLK | 2107.059 | 703.3628 | 32.44 | 4.210905 |
| DVDLQEAYGKGEAGSSEDEEEGGEEGAAA GDQAG | 3326.345 | 832.5944 | 48.78 | 1.607679 |
| VN(+.98)YAGVSTNN(+.98)YALDEVEEVKA | 2286.054 | 1144.039 | 28.5 | 1.759802 |
| S(+42.01)AQ(+.98)LVWELVK | 1214.655 | 608.3331 | 47.77 | 5.003347 |
| LAM(+15.99)LSM(+15.99)FGF | 1047.477 | 524.7496 | 37.52 | 6.481973 |
| TVIWN(+.98)GPM(+15.99)GVFEFDAFSK | 2060.955 | 1031.493 | 41.55 | 2.96499 |
| AVGVVLPHLN(+.98)GK | 1203.698 | 402.2408 | 16.95 | 2.543453 |
| RGGADAVAAGAEER | 1328.643 | 665.3369 | 13.86 | 5.627608 |
| LSELLEGFIK | 1147.649 | 574.8304 | 33.26 | 6.369409 |
| NGVASFGEAVWFK | 1410.693 | 706.3521 | 35.93 | 5.910272 |
| AVAAAETAGSPPK | 1168.609 | 585.317 | 16.86 | 1.258114 |
| LIN(+.98)GILDAAK | 1027.591 | 514.8024 | 22.35 | 2.604158 |

| | | | | |
|---|---|---|---|---|
| LADEITYFLEK | 1340.686 | 671.35 | 26.23 | 4.511042 |
| FEKVNYAGVSTNNYALDEVEEVKA | 2688.292 | 897.1016 | 28.05 | 1.076131 |
| KEEKVEEEEDEDM(+15.99)GF | 1857.746 | 620.2605 | 8.14 | 4.663115 |
| VFPNGEVQYLHPK | 1526.788 | 509.9383 | 17.76 | 2.294477 |
| FIGGFLVK | 879.5218 | 440.7714 | 27.39 | 2.462032 |
| S(+42.01)AQLVWELVK | 1213.671 | 607.8406 | 46.03 | 5.003347 |
| VLEQLTGQ(+.98)QPVFGK | 1543.825 | 772.9136 | 23.18 | 3.334917 |
| GLPALVGLLR | 1007.649 | 504.8331 | 30.1 | 7.372726 |
| VVDDN(+.98)FGIK | 1006.497 | 504.2642 | 15.88 | 3.427976 |
| VFLEN(+.98)VVR | 975.5389 | 488.7787 | 21.93 | 4.298178 |
| TVVELEGVGAGGK | 1214.651 | 608.332 | 20.12 | 2.460017 |
| LYPGGPFDPLGLADDPEVLAELK | 2425.242 | 809.4077 | 47.18 | 3.265296 |
| LQAQAEEQLADAARK | 1640.848 | 821.4326 | 13.59 | 0.815697 |
| ALDKPFAM(+15.99)PVEDVFSIQ(+.98)GR | 2136.056 | 713.0286 | 31.21 | 3.799749 |
| E(-18.01)M(+15.99)SLLLR | 858.4633 | 430.2401 | 21.09 | 5.70411 |
| LGYSDEDLLLPLLDVSVK | 1988.072 | 663.6976 | 47.67 | 5.545578 |
| LIFAFK | 737.4476 | 369.7321 | 25.34 | 2.605729 |
| TSQNSELNNMQDLVEDYK | 2126.943 | 1064.485 | 29.1 | 1.590976 |
| LASYLDKVQ(+.98)ALEEANNDLENK | 2377.165 | 793.3861 | 27.23 | 3.214425 |
| IDGYIYR | 898.4548 | 450.239 | 11.39 | 2.804397 |
| ALVLVGGYGTR | 1104.629 | 553.3224 | 18.15 | 4.532362 |
| GAPAAAAAAPAAGGAAAAEEK | 1692.843 | 847.4302 | 6.85 | 3.444037 |
| TAIAIDAIIN(+.98)QK | 1270.713 | 636.3615 | 26.3 | 1.493575 |
| ELVAQEGTDVN(+.98)EKDDEGR | 2003.892 | 668.9723 | 7.37 | 4.27714 |
| VAIN(+.98)GFGR | 833.4395 | 417.7303 | 14.02 | 6.238151 |
| SELAC(+57.02)VYAVLALADDGLEVTPENIV TLTK | 3103.6 | 1035.536 | 51.62 | 2.310158 |
| LHNNQLEGSIPAEWGQ(+.98)PTAFAR | 2436.182 | 813.0746 | 36.83 | 4.424236 |
| EFLEGLR | 862.4548 | 432.2408 | 19.02 | 4.550042 |
| ITPDDLLR | 941.5182 | 471.7665 | 20.67 | 4.315457 |
| LFDPVYLFDQG | 1312.634 | 657.3214 | 47.78 | -0.25497 |
| LGDEEAGLIGGN(+.98)Q(+.98)LR | 1542.753 | 772.3834 | 23.46 | 8.707296 |
| SWDVFAEILEK | 1335.671 | 668.8441 | 43.53 | 3.975435 |
| ALAIAALLSLR | 1110.712 | 556.3574 | 36.7 | 4.318589 |
