# Supplementary material for: Quantitative Structure-Activity Relationship Model to Predict Antioxidant Effects of the Peptide Fraction Extracted from a Co-Culture System of Chlorella pyrenoidosa and Yarrowia lipolytica
Source: Mar Drugs. 2019 Nov 8;17(11):633. doi: 10.3390/md17110633 (PMC6891513; doi:10.3390/md17110633)
Supplement: Supplementary file 1 [file marinedrugs-17-00633-s001.zip › marinedrugs-624288 suppl/Table S3.pdf]

Table S3. Distribution of peptides with different molecular weights

Molecular weight (Da)	Number of peptides
500-1000	90
1000-1500	393
1500-2000	232
2000-2500	84
2500-3000	20
3000-3600	17
